# Conservatism and Adaptability during Squirrel Radiation: What Is Mandible Shape Telling Us?

**DOI:** 10.1371/journal.pone.0061298

**Published:** 2013-04-04

**Authors:** Isaac Casanovas-Vilar, Jan van Dam

**Affiliations:** 1 Institut Català de Paleontologia Miquel Crusafont, Universitat Autònoma de Barcelona, Cerdanyola del Vallès, Spain; 2 Department of Earth Sciences, Utrecht University, Utrecht, The Netherlands; University College London, United Kingdom

## Abstract

Both functional adaptation and phylogeny shape the morphology of taxa within clades. Herein we explore these two factors in an integrated way by analyzing shape and size variation in the mandible of extant squirrels using landmark-based geometric morphometrics in combination with a comparative phylogenetic analysis. Dietary specialization and locomotion were found to be reliable predictors of mandible shape, with the prediction by locomotion probably reflecting the underlying diet. In addition a weak but significant allometric effect could be demonstrated. Our results found a strong phylogenetic signal in the family as a whole as well as in the main clades, which is in agreement with the general notion of squirrels being a conservative group. This fact does not preclude functional explanations for mandible shape, but rather indicates that ancient adaptations kept a prominent role, with most genera having diverged little from their ancestral clade morphologies. Nevertheless, certain groups have evolved conspicuous adaptations that allow them to specialize on unique dietary resources. Such adaptations mostly occurred in the Callosciurinae and probably reflect their radiation into the numerous ecological niches of the tropical and subtropical forests of Southeastern Asia. Our dietary reconstruction for the oldest known fossil squirrels (Eocene, 36 million years ago) show a specialization on nuts and seeds, implying that the development from protrogomorphous to sciuromorphous skulls was not necessarily related to a change in diet.

## Introduction

The morphology of biological organisms and their anatomical parts is controlled by several factors. Firstly, it may result from the adaptation to specific functions. At the same time it is dependent on phylogeny, which constrains morphology via inheritance. Thus, related taxa will show a tendency to resemble each other more than species drawn at random from the phylogenetic tree [1]–[3]. Importantly, the two factors are not independent, because the phylogeny component includes functional adaptations that took place in ancestors. The third factor includes structural processes that constrain morphology via other variables, such as allometric scaling with body size [4]–[6].

The treatment of phylogenetic effects is now looked upon as an almost compulsory part of morphophunctional analyses above the species level. For instance, comparative methods have been devised that are able to deal with the statistical biases on functional-morphological relationships stemming from phylogenetically affected correlation structures [1]–[3], [7]. Also, the modern toolkit of geometric morphometrics now includes methods and software that address phylogenetic effects [8]–[9].

Because of their distinct and variable shape, mammal mandibles in general have proven to be very suitable for geometric morphometric analysis, both landmark-based [10]–[13] and outline-based [14]–[16]. Here we focus on mandibles of recent squirrels, one of the oldest and most diverse rodent families [17] showing widely differing mandibular morphologies. According to molecular phylogenetic reconstructions, the diversification of squirrels into their main extant clades occurred very early in their history [18]. These results appear to fit known paleontological data: for example, the postcranial skeleton of *Douglassciurus jeffersoni* is so similar to that of the tree squirrel *Sciurus* that the extant species could be considered a ‘living fossil’ [19]–[20]. The following question is therefore justified: is squirrel diversification simply reflecting the near worldwide expansion of a generalized, conservative model, or it can be explained by morphological adaptation to exploit different environments and resources?

Squirrel mandibles have been analysed before using landmark-based geometric morphometrics by Swiderski and Zelditch [21] and outline analysis [16], [22]. These studies have tended to focuss in particular aspects of mandibular shape (i.e., dwarfism [22] or scaling of the mandible lever arms [21]). Here we take a comprehensive landmark-based geometric morphometric approach towards the squirrel mandible that differs from previous works [16] because it explicitly makes use of specific methods that allow for the testing and quantifying the phylogenetic signal in morphometric data, and that map shape changes onto the phylogeny. Additionally, we explore the role of allometric trends. Finally, the biomechanical performance of the different mandible shapes is studied and related to diet.

## Materials and Methods

### Material and shape analysis

The studied material consists of 301 adult mandibles belonging to 44 different species and genera (see Table S1 for a detailed material list). The dataset adequately covers squirrel diversity, including members of all subfamilies and tribes. Genera that are not included are restricted to a number of certain rare flying squirrels (such as *Biswamoyopterus* and *Eoglaucomys*) and various new genera of marmotines that have resulted from the recent splitting of the genus *Spermophilus* into up to 7 different genera on the basis of molecular and morphological data [23]. The extant specimens are stored at the NCB Naturalis (Leiden, the Netherlands) and the Muséum National d′Histoire Naturelle (Paris, France). A fossil mandible of *Douglassciurus jeffersoni* (Eocene, Montana) is also included in some of the analyses. It was obtained from published images of specimen USNM 214936 [24] stored at the National Museum of Natural History, Smithsonian Institution (Washington, USA).

**Figure 1:**
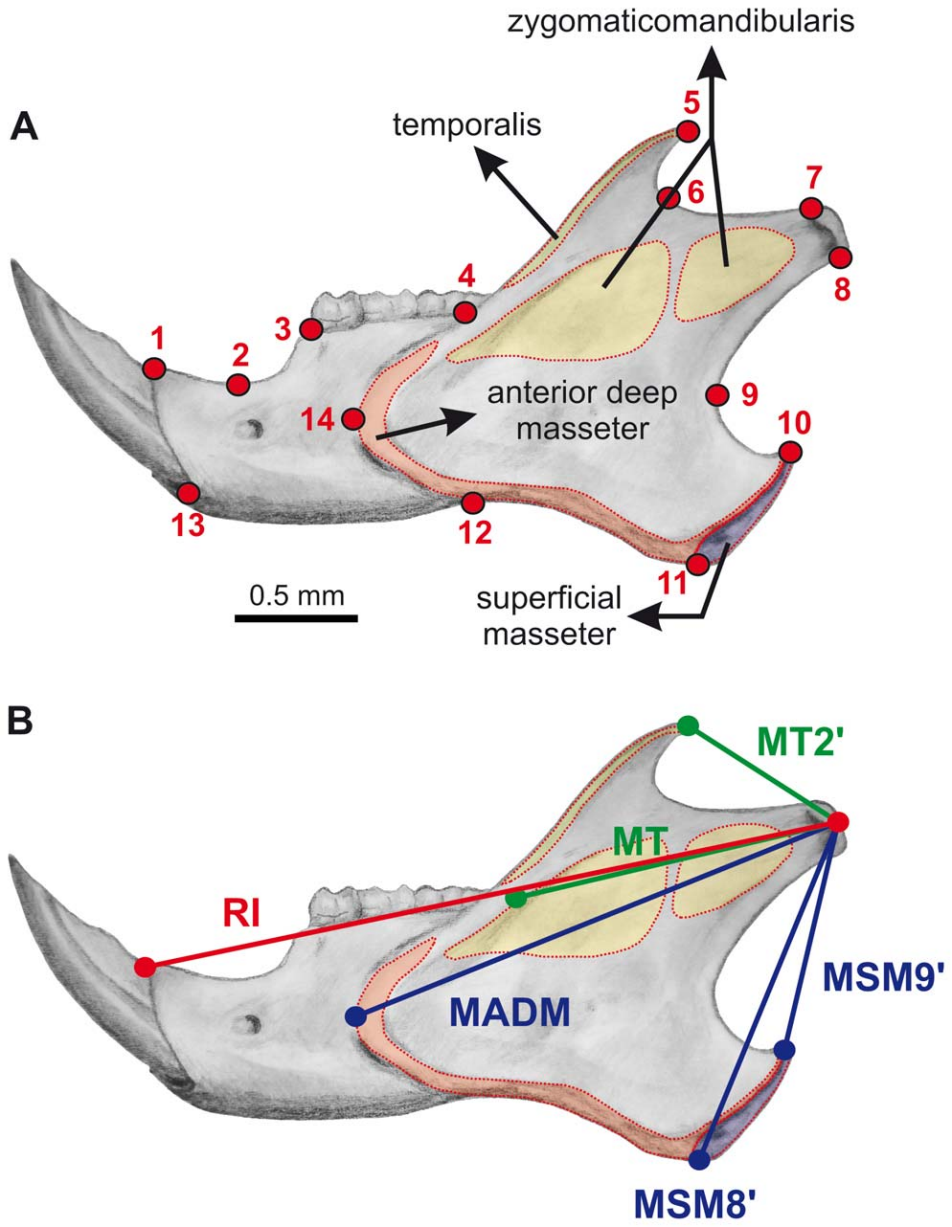
Mandible *Sciurus vulgaris* in lateral view showing landmarks, insertion areas of the masticatory muscles and muscle moment arms. a) Landmarks used in this study: (1) antero-dorsal border of the incisive alveolus; (2) most concave point of the diastema; (3) anterior alveolus of the lower premolar; (4) base of the coronoid process; (5) tip of the coronoid process; (6) most concave point of the incisura mandibulare; (7) anterior edge of the articular surface of the condyle; (8) posterior edge of the articular surface of the condyle; (9) most anterior point on the curve of the posterior edge of the mandible; (10) posterior tip of the angular process; (11) most ventral point of the angular process; (12) most dorsal point on the ventral border of the ramus; (13) antero-ventral border of the incisive alveolus; (14) anterior edge of the masseteric ridge. b) Muscle moment arms and resistance arm for the incisor. MSM9′  =  moment arm of the most dorsally inserting fibers of the superficial masseter; MSM8′  =  moment arm of the most ventral fibers of the superficial masseter; MADM  =  moment arm of the most anterior fibers o the anterior deep masseter; MT  =  moment arm of the most ventral fibers of the temporalis; MT2′  =  moment arm of the most dorsal fiber of the temporalis; RI  =  resistance arm of the incisor. See text for details.

All specimens were digitally photographed in buccal view. Mandibles were oriented with the corpus parallel to a horizontal plane. A total of 14 landmarks were defined were selected so that they adequately describe shape and capture functional key structures of the mandible (Fig. 1a). Most of them correspond to type 2 landmarks in the classification of Bookstein [25]. The landmarks. Landmark coordinates were obtained using the TPSDig2 software [26], while all subsequent shape analyses were carried out using MorphoJ [27]. A Generalized Procrustes Analysis (GPA) [25] was conducted on the raw coordinates to remove the effects of translation, rotation and scale, thereby superimposing all specimens and allow the derivation of a consensus shape configuration. Size in the form of centroid size (CS) was stored for all specimens. Mean per-taxon shapes were computed by averaging mandible shapes for all specimens within a single genus. To display the arrangement of the data points, we used principal component analysis (PCA) of the covariance matrix of shape tangent coordinates.

### Testing for evolutionary allometry

A multivariate regression of Procrustes coordinates (PRC) on centroid size (CS) was performed in order to investigate shape variation with size. CS is the only size measure truly grounded in geometric morphometrics theory [21], [28]–[31]. The proportion of shape variation predicted by size was determined using the Procrustes metric [32]. Additionally we conducted separate regression analyses for each of the main squirrel subfamilies and tribes in order to search for size trends within these groups. Differences in slope and intercept were tested using ANCOVA [30]. Species mean values were used in all those calculations instead of individual specimens. In the case a strong correlation between size and shape was detected, a covariance matrix using the residuals of the regression was built and used in subsequent analyses.

### Mapping shape variation onto phylogeny and quantifying phylogenetic signal and homoplasy

Phylogenetic trees can be projected into a morphometric space in order to visualize the evolutionary history of morphometric traits [9], [33]–[35]. On the other hand, morphometric data can be projected onto a phylogenetic tree and ancestral shapes can be reconstructed for the internal nodes using squared-shape parsimony [36]. We have conducted both approaches with MorphoJ [27] using the published phylogeny of the Sciuridae after Mercer & Roth [18]. The phylogenetic tree was imported into MorphoJ from a NEXUS file built with Mesquite [37] without taking into account branch lengths. We used the molecular phylogeny of Mercer & Roth [18] instead of the more recent one by Steppan *et al*. [38] because the former includes a greater number of genera and has been considered in a previous study of squirrel mandible shape [16]. The two phylogenies differ in a few details only, pertaining mainly to the position of the Asian rock squirrel (*Sciurotamias*), which lies outside the Protoxerini/Marmotini clade in Mercer & Roth [18], whereas it is placed within the Marmotini in Steppan *et al*. [38].

We used a method devised by Klingenberg & Gidaszewski [9] to test for the presence of phylogenetic signal in morphometric data. The test simulates the null hypothesis of total absence of phylogenetic structure by permuting shape data among the terminal taxa in a given phylogeny. It is a highly conservative test that will reject the null hypothesis of independence if a strong phylogenetic signal is present in only one or few clades even though it is absent in all the remaining ones. Because of this property we also split the data according to subfamily (Sciurinae, Xerinae and Callosciurinae) and repeated the test within each of these clades (the remaining subfamilies Sciurillinae and Ratufinae are monotypic and therefore not suitable for such analyses.) Whereas the permutation test evaluates the presence or absence of a phylogenetic signal, it does not quantify its strength. In order to do so, we have calculated the shape consistency (SCI) and shape retention indices (SRI) [9]. These indices are analogous to the retention and consistency indexes used in cladistic analysis. Values are always positive and range from 0 to 1, with higher values indicating lower degrees of homoplasy. The calculation of both indexes requires that tree length is known as well as the maximal and minimal length of any tree fitting the set of shapes of the terminal taxa [9]. The calculation of the tree with minimal length is a complicated and much-debated issue known as the Steiner tree problem [39]. Effective algorithms for finding squared-change Steiner trees for multidimensional data have been developed [39] but only work with small numbers of taxa (less than twelve) [9]. In our case this means that SCI and SRI could only be calculated for the Callosciurinae. The permutation test for the presence of phylogenetic signal as well as the calculation of observed and maximum tree length was performed using MorphoJ [27]. Minimum tree length was computed using the FindSteinerTree software [9].

### Ecomorphology

Squirrel species were assigned to different groups according to their locomotory and dietary preferences (see Table S1). We left ungrouped the extinct *Douglassciurus jeffersoni* as well as certain extant flying squirrel species for which dietary preferences are not well known. Differences among groups were explored using Canonical Variates Analysis (CVA) of Procrustes coordinates. Instead of genus means all specimens were considered separately in CVA because otherwise very small sample sizes would result for some of the dietary groups. Dietary groups are qualitative and include up to seven categories: 1) frugivores; 2) nut eaters; 3) granivores; 4) folivores; 5) herbivores *sensu stricto*, i.e. feeding on grasses and/or other hard tissues of plants; 6) bark gleaners, i.e. squirrels that feed by grasping and yanking fragments of bark; 7) insectivores. Procrustes distances between all these groups were computed. The patterns of shape variation were compared by matrix correlation using the MorphoJ software and assessed statistically with matrix permutation tests specifically adapted for geometric morphometric data [27].

It should be noted that many squirrels are in fact omnivorous, with many species showing seasonal and geographical dietary variation, particularly in temperate seasonal climates. Therefore, assignment to a single category is sometimes hazardous. As this is a mandatory requirement for CVA, we grouped the species according to the staples in their diet (see Table S1), and discuss seasonal/geographical variations *a posteriori*. In order to compare our results with previous studies on squirrel mandible shape, such as that of Michaux *et al*. [16], we also conducted a separate CVA using their broader dietary categories as well as their locomotory categories.

Additionally, we measured the resistance arm for the incisor and the moment arms for the main masticatory muscles [40]–[41] (see Fig. 1b and Table 1). The resistance arm for the incisor was not measured at its tip because this point showed a large degree of variation due to wear. Instead we calculated the incisor resistance arm at the antero-dorsal border of its alveolus. This implies a somewhat shorter resistance arm, but in all cases it is longer that any muscle moment arm. Moment arms were measured from the tip of the mandibular condyle (i.e. the midpoint between landmarks 7 and 8; Fig. 1b) to the landmarks representing the extreme points of the muscular insertions (Fig. 1b). The measurement at the insertion of the temporalis immediately posterior to the third molar was taken using a calliper, because the ramus covers this area in lateral view. The mechanical advantage of the jaw muscles, i.e., a measure of force amplification by a particular muscular arrangement, was evaluated considering the resistance arm at the incisor alveolus which would relate to the power stroke during incisor bite.

**Table 1:** Mechanical advantage of main mandibular muscles expressed as ratio of moment arms to incisor resistance arm.

		Temporalis			Superficial masseter			Anterior deep masseter
Species	Dietary preference	MT2′		MT	MSM8′	MSM9′		MADM
*Aeromys tephromelas*	fruits	0.21		0.49	0.56	0.41		0.68
*Ammospermophilus leucurus*	herbivore s.s.	0.23		0.52	0.54	0.31		0.75
*Atlantoxerus getulus*	seeds	0.23		0.49	0.50	0.36		0.74
*Belomys perasonii*	leaves	0.21		0.44	0.56	0.38		0.70
*Callosciurus erythraeus*	fruits	0.26		0.57	0.54	0.38		0.76
*Cynomys ludovicianus*	herbivore s.s.	0.23		0.40	0.51	0.34		0.75
*Dremomys rufigenis*	insects	0.21		0.55	0.50	0.37		0.77
*Epixerus ebii*	nuts	0.26		0.55	0.54	0.40		0.78
*Eupetaurus cinereus*	herbivore s.s.	0.27		0.44	0.59	0.39		0.71
*Exilisciurus exilis*	bark gleaner	0.31		0.54	0.54	0.36		0.81
*Funambulus palmarum*	nuts	0.32		0.55	0.50	0.36		0.78
*Funisciurus congicus*	nuts	0.21		0.54	0.56	0.40		0.76
*Glaucomys volans*	nuts	0.22		0.56	0.55	0.37		0.78
*Heliosciurus gambianus*	fruits	0.21		0.55	0.57	0.41		0.76
*Hylopetes lepidus*	unknown	0.22		0.53	0.58	0.37		0.73
*Iomys horsfieldii*	fruits	0.24		0.53	0.55	0.30		0.72
*Lariscus insignis*	fruits	0.24		0.55	0.52	0.35		0.75
*Marmota marmota*	herbivore s.s.	0.21		0.48	0.45	0.29		0.76
*Menetes berdmorei*	seeds	0.28		0.52	0.48	0.33		0.71
*Microsciurus flaviventer*	insects	0.27		0.55	0.56	0.38		0.80
*Myosciurus pumilio*	bark gleaner	0.33		0.50	0.50	0.39		0.87
*Nannosciurus melanotis*	bark gleaner	0.30		0.54	0.56	0.38		0.79
*Paraxerus ochraceus*	leaves	0.19		0.52	0.54	0.38		0.73
*Petaurista petaurista*	fruits	0.23		0.53	0.57	0.41		0.75
*Petaurillus kinlochii*	unknown	0.24		0.60	0.58	0.38		0.81
*Petinomys genibarbis*	unknown	0.23	0.53		0.57		0.35	0.74
*Prosciurillus leucomus*	fruits	0.29	0.58		0.57		0.39	0.79
*Protoxerus stangeri*	nuts	0.26	0.57		0.57		0.39	0.78
*Pteromys volans*	leaves	0.18	0.48		0.66		0.46	0.84
*Pteromyscus pulverulentus*	unknown	0.15	0.53		0.47		0.29	0.72
*Ratufa bicolor*	nuts	0.24	0.59		0.63		0.44	0.82
*Rheithrosciurus macrotis*	fruits	0.28	0.68		0.59		0.41	0.89
*Rhinosciurus laticaudatus*	insects	0.28	0.45		0.40		0.31	0.64
*Sciurillus pusillus*	bark gleaner	0.32	0.60		0.57		0.41	0.88
*Sciurotamias davidianus*	seeds	0.26	0.52		0.49		0.31	0.70
*Sciurus vulgaris*	nuts	0.25	0.55		0.54		0.38	0.75
*Spermophilopsis leptodactylus*	herbivore s.s.	0.23	0.43		0.52		0.28	0.79
*Sundasciurus altitudinis*	seeds	0.21	0.56		0.55		0.38	0.76
*Tamias stritatus*	seeds	0.23	0.50		0.47		0.31	0.70
*Tamiasciurus hudsonicus*	nuts	0.22	0.56		0.54		0.39	0.81
*Tamiops mcclellandii*	insects	0.24	0.55		0.51		0.35	0.75
*Trogopterus xantiphes*	leaves	0.23	0.39		0.52		0.37	0.71
*Urocitellus undulatus*	herbivore s.s.	0.24	0.44		0.46		0.29	0.72
*Xerus erythropus*	seeds	0.26	0.49		0.49		0.32	0.73
*Douglassciurus jeffersoni*	unknown	0.23	0.45		0.60		0.37	0.73

See Figure 1 and main text for details.

MT2′  =  moment arm of the most dorsal fiber of the temporalis; MT  =  moment arm of the most ventral fibers of the temporalis; MSM9′  =  moment arm of the most dorsally inserting fibers of the superficial masseter; MSM8′  =  moment arm of the most ventral fibers of the superficial masseter; MADM  =  moment arm of the most anterior fibers o the anterior deep masseter.

## Results

### Evolutionary allometry

**Figure 2:**
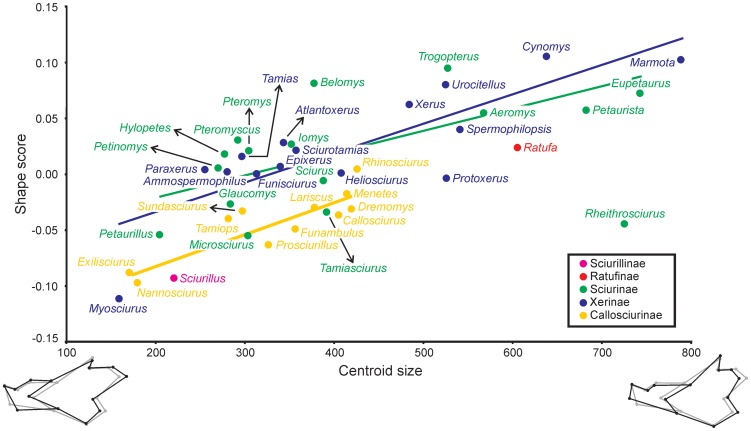
Shape change associated with evolutionary allometry estimated from regression of shape on centroid size. Regression analyses for each one of the squirrel subfamilies. Differences in the intercept and slope are tested using ANCOVA (see text for details). Only genera means are shown.

The multivariate regression of Procrustes coordinates (PRC) on centroid size (CS) is highly significant (*F*_1, 42_ = 32.18; *P*<0.001), accounting for 12.93% of total shape variation (Fig. 2). The correlation of PRC with CS indicates that there is allometry in shape change, even though only a small percent of shape variation is explained by size. Allometric changes affect all regions of the mandible. Smaller mandibles tend to be short and to have somewhat reduced angular and coronoid apophyses, whereas the articular apophysis is elongated and projecting backwards. The masseteric turbercle, marking the end point of the masseteric ridge, is displaced more anteriorly in small specimens (Fig. 2). In contrast, larger squirrels show a considerably elongated mandible with a reduced articular process. The coronoid process is not reduced, whereas the angular one is somewhat expanded (Fig. 2). This has implications for the mechanical advantage of the mandibular muscles (Table 2). Specifically, the mechanical advantage for the anterior deep masseter and the most ventral fibers of the temporalis is greater in small-sized squirrels, whereas there are almost no size-related changes in the performance of all other muscles.

**Table 2:** Mechanical advantage of main mandibular muscles expressed as ratios of moment arms to incisor resistance arm for mean shapes of dietary groups.

Shape	Temporalis		Superficial masseter		Anterior deep masseter
	MT2′	MT	MSM8′	MSM9′	MADM
Frugivores mean	0.23	0.56	0.55	0.38	0.77
Nut eaters mean	0.23	0.56	0.56	0.40	0.79
Granivores mean	0.24	0.51	0.50	0.36	0.74
Folivores mean	0.22	0.47	0.57	0.41	0.72
Herbivores s.s. mean	0.22	0.44	0.49	0.31	0.73
Bark gleaners mean	0.32	0.56	0.55	0.38	0.84
Insectivores mean	0.25	0.52	0.50	0.35	0.74
Centroid size = 100 mm	0.25	0.60	0.53	0.38	0.81
Centroid size = 800 mm	0.23	0.45	0.54	0.37	0.72
PC1 score = −0.20	0.34	0.51	0.45	0.31	0.76
PC1 score = 0.15	0.22	0.54	0.59	0.40	0.77
PC2 score = −0.15	0.21	0.40	0.45	0.30	0.66
PC2 score = 0.15	0.28	0.64	0.45	0.45	0.85
CV1 score = −6.0	0.22	0.41	0.51	0.35	0.69
CV1 score = 6.0	0.30	0.61	0.56	0.43	0.82
CV2 score = −6.0	0.30	0.48	0.48	0.30	0.79
CV2 score = 6.0	0.22	0.58	0.58	0.43	0.73

**Figure 3:**
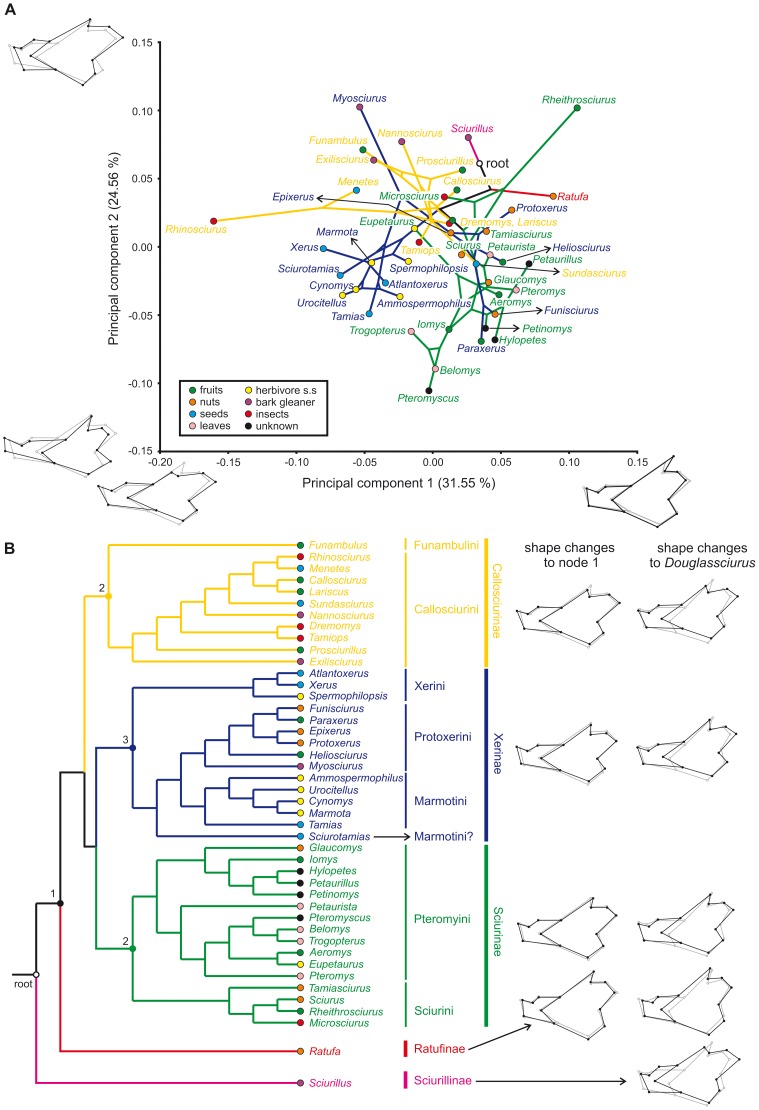
Principal Component Analysis (PCA) of mandible shape, squirrel phylogeny and dietary preferences. (a) Size-corrected PCA of covariance matrix among species means with phylogenetic tree [18] projected onto the shape space defined by the first two principal components. Black mandible outlines along the axes represent shape changes associated with each principal component; grey outlines represent the consensus configuration; (b) Squirrel phylogeny used [18]. Subfamily colours correspond to colour pattern used in the PCA. Colours of terminal points indicate dietary preferences (also used in PCA and CVA, see Fig. 4, Figs. S1–S4). Right part of the figure shows morphometric changes along the tree starting from the reconstructed ancestral shape at node 1 and from that of the oldest-known squirrel, *Douglassciurus jeffersoni* (grey outlines). Black mandible outlines show the reconstructed ancestral shape for each squirrel subfamily (node 2: Sciurinae; node 3: Xerinae; node 4: Callosciurinae).

**Figure 4:**
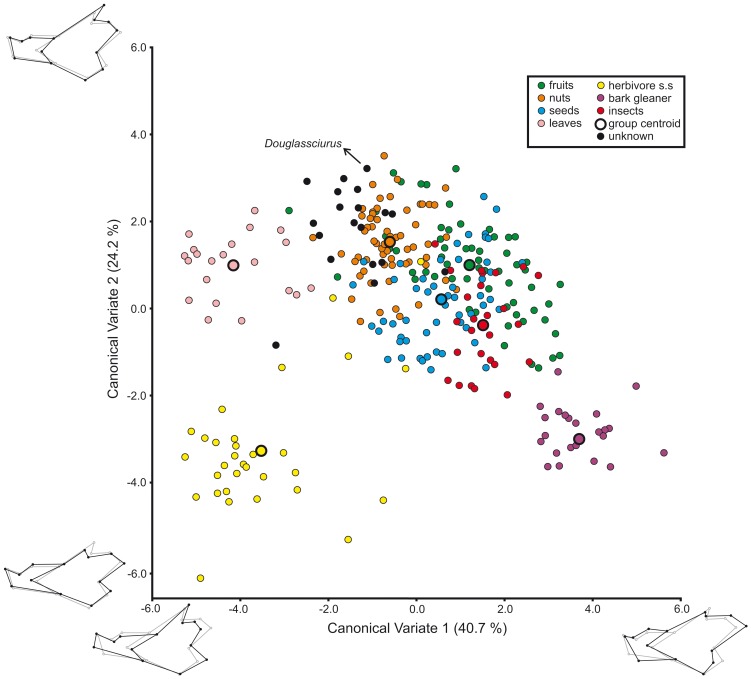
Canonical Variates Analysis (CVA) of squirrel mandible shape using dietary preferences as grouping variable. Plot of CV1 and CV2. For CV3 and CV4: see Figure S4. For a summary of classification results: see Table 3. For the results for each particular case: see Table S3.

We also include the same calculations for reconstructed shape changes along the main PCA axes Analysis (Fig. 3); along the main CVA axes (Fig. 4); and for the regression of shape on centroid size (Fig. 2). Lever arm for most ventral fibers of the temporalis (MT) of reconstructed shapes had to be calculated as the distance between the tip of articular process and landmark 4 (see Fig. 1).

MT2′  =  moment arm of the most dorsal fiber of the temporalis; MT  =  moment arm of the most ventral fibers of the temporalis; MSM9′  =  moment arm of the most dorsally inserting fibers of the superficial masseter; MSM8′  =  moment arm of the most ventral fibers of the superficial masseter; MADM  =  moment arm of the most anterior fibers o the anterior deep masseter.

If the analyses are repeated for each subfamily separately, there is a significant relationship between CS and PRC in the Callosciurinae and the Xerinae (*F*_1, 9_ = 27.90; *P* = 0.001 and *F*_1, 13_ = 27.19; *P*<0.001, respectively), whereas mandible shape in the Sciurinae is not correlated with size (*F*_1, 14_ = 2.71; *P* = 0.12). This is reflected in Fig. 2 where the regression line for the Sciurinae has a slightly more horizontal slope than that of the Xerinae and the Callosciurinae. Despite these apparent between-subfamily differences, ANCOVA results indicate that there are no significant differences in the slope (*F*_2_ = 2.19; *P* = 0.126) or intercept (*F*_2_ = 2.78; *P* = 0.075) between the three groups. Similar to Hautier *et al*. [20], we conducted the same analyses at the tribe level. Once more, the analyses do not detect significant differences in the slope (*F*_5_ = 1.52; *P* = 0.213) or the intercept (*F*_5_ = 1.805; *P* = 0.142). Therefore, our results indicate that the relationship of shape with size is similar in all squirrel subfamilies and tribes.

### Shape variation

Even though only a small percent of shape variation is accounted by size differences we decided to use the residuals of regressions of PRC on CS as the input for a PCA (Fig. 3a). The principal components (PC) are therefore uncorrelated with CS. The first four PCs account for about 74% of the variation: PC1 explains 31.55% of the variation among species means, PC2 24.56%, PC3 10.01% and PC4 7.19%. Each one of the subsequent PCs accounts for less than 5%. PC1 represents variation between a low mandible with a well-developed, projecting articular process to a markedly reduced coronoid one to a high mandible with a long, postero-dorsally directed coronoid and a relatively wider angular process. Squirrels with elongated articular processes score negatively on this axis, particularly the insectivorous *Rhinosciurus*. To a lesser degree, a long articular process is also present in most ground squirrels of the tribes Xerini and Marmotini as well as in many Callosciurinae (*Menetes*, *Nannosciurus*, *Exilisciurus*, *Funambulus*) and the dwarf Protoxerini *Myosciurus*. On the other hand, many Sciurinae species, cluster altogether at moderate to high values because they posses higher mandibles with well-developed coronoid processes. PC2 mostly expresses changes in mandible elongation, position of the masseteric ridge and the relative development of the processes. At high values mandibles are markedly short and exhibit a robust corpus, besides a well-developed articular process at the expense of the angular one, and an anteriorly-placed masseteric ridge. At the other end of the axis the opposite patterns occur, with mandibles being elongated and low with a reduced and narrow articular process and a more projecting angular process, and the masseteric ridge moving posteriorly. *Myosciurus* and *Rheithrosciurus* show extreme positive values, whereas they are positioned at opposite sides of the PC1 axis. Both genera show short and robust mandibles with a forward-positioned strong masseteric ridge and a wide articular process, which is long in *Myosciurus* and short in *Rheithrosciurus*. Additionally, the coronoid apophysis is extremely reduced in the former genus, while in the second one it is comparable to that of other large-sized tree squirrels such as *Ratufa*. Most Callosciurinae, possessing a projecting articular process and a reduced coronoid one, also score high on this axis. Lower values are reserved for ground squirrels as well as certain flying squirrels (*Trogopterus*, *Pteromyscus*, *Belomys*, *Hylopetes*), which are characterized by elongated mandibles with usually narrow articular processes and long angular ones. Finnally, PC3 (not shown) discriminates the woolly flying squirrel (*Eupetaurus cinereus*) from all the other species because of its unique long mandible with a high ramus.

The arrangement of the specimens in the plane of the first two PCs suggests a relationship between mandible shape and ecology. The mechanical advantages of all muscles but that of the most dorsal fibers of the temporalis increase along PC1 (Fig. 3, Table 2). Most of the flying and tree squirrels that feed on fruits and nuts have high positive values on PC1, which is consistent with their powerful muscles providing a strong incisor bite. *Rheithrosciurus*, which is characterized by stout, massive incisors, shows the highest values. On the other hand, negative values mostly characterize squirrels that feed on seeds, herbs or insects and show lower mandibles and more reduced coronoid processes. The mechanical advantages of most mandibular muscles increase along PC2, whereas for the most ventral fibers of the superficial masseter it remains unchanged (Fig. 3, Table 2). The mechanical advantages of the most ventral fibers of the temporalis and the anterior deep masseter show a more marked increase along this axis compared to other muscles. This increase is due to the lengthening of the articular process and the forward displacement of the masseteric ridge, respectively. These features are characteristic of many small squirrels that glean bark, as well as the frugivore *Rheithrosciurus*. At the other end of the axis we find the elongated mandibles of many ground squirrels and certain flying squirrels. Most of those squirrels are granivores, but some of them have specialized in consuming leaves (*Trogopterus*, *Belomys*) or even grasses (*Cynomys*, *Urocitellus*, *Marmota*).

### Quantifying phylogenetic signal and homoplasy

We projected squirrel phylogeny (Fig. 3b) onto the shape space defined by the first two PCs using different colors for each squirrel subfamily (Fig. 3a). Apparently there is a significant degree of phylogenetical signal in the shape data, since related taxa tend to occupy particular regions in multivariate space. Virtually all the ground squirrels of the Xerini and Marmotini cluster close to one another on the first three PCs. To a lesser degree this is also observed for the Pteromyini and the Callosciurinae, although certain uniquely-shaped taxa, such as *Rhinosciurus* or *Eupetaurus*, plot separate from the group that includes most of their closest relatives. The permutation test of shape data among the terminal taxa confirms the impression that there is phylogenetic structure in the data (Tree length = 0.146; *p* (no signal)<0.0001). As noted before, if a strong phylogenetic signal would have been present in just one or a few clades, the test would find a strong phylogenetic structure even if it is absent in all other clades. When the data are split into three major clades (Sciurinae, Xerinae, Callosciurinae; Figs. S1–S3), *p* (no signal) is 0.0005 for the Xerinae and 0.0009 for the Sciurinae. Only for the Callosciurinae the null hypothesis of complete absence of phylogenetic structure in the data cannot be rejected (*p* = 0.284). This is probably due to the presence of taxa with highly specialized morphologies within this clade such as the ‘anteater’ squirrel *Rhinosciurus* and the bark gleaner *Nannosciurus* (Fig. S3). Shape retention (SRI) and shape consistency (SCI) indexes could only be calculated for the Callosciurinae. The measures take very high values (SCI = 0.800; SRI = 0.994) and indicate a high degree of synapomorphy and a low degree of homoplasy. Even if we cannot reject the null hypothesis of phylogenetic structure in the data, the indexes suggest a strong phylogenetic signal in the Callosciurinae as well.

The mapping of morphometric changes along the phylogenetic tree is shown in Fig. 3b and includes the reconstruction of the ancestral shapes at the root as well as at the branching point in each subfamily (Fig. 3b). The reconstructed ancestral (root) mandible is short and high and contains a projecting articular process and reduced coronoid and angular processes. The shape differs markedly from that of the oldest-known squirrel, *Douglassciurus jeffersoni*. The reason for this difference is that the ‘root’ shape is highly influenced by the phylogenetic position of *Sciurillus* (subfamily Sciurillinae), which branched off very early in squirrel phylogeny and evolved a unique mandible adapted to bark gleaning (see discussion below). The reconstructed shape at node 1 (Fig. 3b) is ancestral to all other extant squirrel genera and is more similar to that of *Douglassciurus*. This ‘ancestral mandible’ is relatively high, without reduction or elongation of any of the mandibular processes, something that occurs in many squirrel genera (Fig. 3b). The mandible of *Douglassciurus* itself is higher than this ‘ancestral mandible’ with the masseteric ridges placed more posteriorly and the coronoid process being relatively longer.

The shapes of the monotypic Ratufinae, which diverged earlier than all other squirrels except for *Sciurillus*, are similar to the reconstructed ancestral shape. They are also closer to that of *Douglassciurus* than other subfamilies, although they differ by their shorter mandible, with a more anteriorly placed masseteric ridge and a longer coronoid process and a broader angular process. The reconstructed ancestral shapes for the Xerinae, Sciurinae and Callosciurinae are remarkably similar to one another and do not depart much from the reconstructed shape at node 1. The three subfamilies differ in the elongation of the mandible as well as in the development of their mandibular apophyses. The ancestral shape of the Sciurinae shows a relatively short and high mandible and is closest to that of node 1. The shape of the Xerinae is relatively elongated, with slightly narrower coronoid and articular processes, whereas the angular process has moved dorsally. The ancestral shape for the Callosciurinae diverges most from the root shape. As in the Xerinae, the mandible is quite elongated, but all processes are narrower. Notably the articular process is long, while the coronoid is reduced and placed more anteriorly. The reconstructed ancestral shapes for the three subfamilies differ considerably from that of *Douglassciurus*. Specifically, the masseteric ridges are placed in a more distinctly anterior position. Furthermore, the mandibles are lower, with a narrower angular process and a relatively longer articular one. Finally, in the Callosciurinae the coronoid process is markedly reduced as compared to that in *Douglassciurus*.

### Ecomorphology

A CVA was carried out with seven dietary groups (Fig. 4, Table 3). Specialized groups such as folivores, insectivores or bark gleaners include between 20 and 30 specimens belonging to a few species only. On the other hand, the frugivores, nut eaters and granivores include about 60 different specimens each representing numerous different species. Some flying squirrels (*Hylopetes*, *Petaurillus*, *Petinomys*, *Pteromyscus*) for which the feeding preferences are not well known were left ungrouped. The fossil *Douglassciurus jeffersoni* was also left ungrouped.

**Table 3:** Summary of the classification results for the Canonical Variates Analysis (CVA) of squirrel mandible shape using dietary preferences as grouping variable.

	Fruits	Nuts	Seeds	Leaves	Herbivore s.s.	Bark gleaner	Insects
**Fruits**	87.10(77.42)	6.45(6.45)	1.61(8.06)	1.61(1.61)	0.00(0.00)	0.00(0.00)	3.22(6.45)
**Nuts**	6.67(8.33)	91.67(86.67)	1.67(3.33)	0.00(1.66)	0.00(0.00)	0.00(0.00)	0.00(0.00)
**Seeds**	19.64(21.43)	0.00(1.78)	75.00(69.64)	0.00(0.00)	0.00(0.00)	0.00(3.57)	5.36(3.57)
**Leaves**	0.00(0.00)	0.00(9.52)	0.00(0.00)	100.00(90.48)	0.00(0.00)	0.00(0.00)	0.00(0.00)
**Herbivore s.s.**	0.00(0.00)	3.23(3.23)	6.45(6.45)	3.23(3.23)	87.10(83.87)	0.00(0.00)	0.00(3.23)
**Bark gleaner**	0.00(0.00)	0.00(0.00)	0.00(0.00)	0.00(0.00)	0.00(0.00)	100.00(100.00)	0.00(0.00)
**Insects**	22.22(37.04)	0.00(3.70)	3.70(3.70)	0.00(0.00)	0.00(0.00)	3.70(7.41)	70.37(48.15)

The numbers refer to the percent of cases assigned to each category. The results after cross-validation are in brackets.

The analysis shows significant differences between all the dietary groups (Table S2) and correctly classifies 86.2% of the cases and 78.7% after cross-validation (leaving the target specimens out, Table 3). The dietary groups are also found to be significantly different if genera means are used instead individual specimens (results not shown). The majority of miss-classified specimens either belongs to the granivore or insectivore category (see Table 3; for probabilities of group membership and discriminant scores for each specimen see Table S3). Most miss-assignments refer to just a few specimens within a species. Cases in which all the specimens of a given species are miss-classified are very rare. Some of the wrong assignments are easy to understand. For example, 8/10 specimens of *Sundasciurus altitudinis* are wrongly assigned to the frugivores instead of the granivores. Even though this species is reported to feed on acorn-like nuts [42] its diet is not adequately known, and many other species within the same genus commonly feed on fruits [43]. Something similar may have happened in the case of *Menetes berdmorei*: 6/10 specimens are wrongly classified as either frugivores or insectivores instead of granivores. *M. berdmorei* is known to enter corn and rice fields in order to dig up and eat planted grain [44], but there is no information available on its dietary preferences in the wild [43]. Finally, certain specimens are wrongly assigned to groups that feed on items they only secondarily consume. For example, *Ammospermophilus leucurus* is wrongly assigned to the granivores. Seeds are an important component in the diet of this species, which nevertheless feeds heavily on green vegetation [45]. *Tamiops macclellandii* and *Funisiciurus congicus* represent similar cases. Finally, the rare woolly flying squirrel *Eupetaurus cinereus* is wrongly classified in all the analyses. This bizarre hypsodont flying squirrel feeds almost exclusively on pine needles [46] and it is wrongly classified as a folivore. In this case the mandible still reflects the shared ancestry with other flying squirrels such as *Belomys*, *Pteromys* or *Trogopterus*, which preferentially feed on green leaves.

With moderate to high probability (see Table S3), the ungrouped flying squirrel species were classified as either feeding on fruits or nuts. On the other hand, *Douglassciurus jeffersoni* was assigned to the group feeding on nuts with a high probability (*p* = 0.904).

The first canonical variate (CV1) accounts for 40.7% of total variance and separates certain folivore (such as *Trogopterus* or *Belomys*) and herbivore squirrels (such as *Marmota*, *Cynomys* or *Urocitellus*) from bark gleaners (*Nannosciurus*, *Exilisiciurus*, *Myosciurus* and *Sciurillus*) with species feeding on nuts, fruits, seeds or insects occupying a wide area between these extremes (Fig. 4). CV1 basically reflects the change in the relative importance of the mandibular processes and the depth of the corpus. At the positive end mandibles show a deep corpus with a well-developed articular apophysis at the expense of all the other processes (as is typical of bark gleaners). Negative values are attained for elongated mandibles characterized by long and broad coronoid and angular apophyses. The axis also reflects changes in the position of the anterior end of the masseteric ridge, which is characteristically placed mesially in bark gleaners. Finally, it also shows the dorsal displacement of the angular process, as typical of ground squirrels, especially those feeding on hard plant tissues. The mechanical advantages of all the mandibular muscles increase along CV1 (see Tables 1–2). The bark gleaners do show the highest values, implying that the incisor bite is particularly strong in these squirrels. By contrast, herbivore and certain folivore and granivore squirrels differ from all other groups by their lower mechanical advantages for all muscles.

CV2 accounts for 24.2% of the variance and basically distinguishes herbivores and bark gleaners from all the remaining squirrels but not from one another (Fig. 4). The mandibles of those two groups show a ramus that is generally lower than that of other squirrels as well as a somewhat reduced and anteriorly-placed coronoid process. Similar morphologies also occur in certain insectivorous (*Rhinosciurus*, *Tamiops*) and granivorous (*Sciurotamias*, *Xerus*, *Menetes*) squirrels, as reflected by negative values on CV2. The remaining squirrels occupy a wide area showing the more ‘standard’, deeper mandibles with a well-developed coronoid process (Fig. 4). CV2 mainly reflects differences in the mechanical advantage of the anterior deep masseter and the most dorsal fibers of the temporalis at the expenses of the most ventral fibers of this muscle and the superficial masseter (see Tables 1–2). The mechanical advantage of the superficial masseter increases along CV2, with bark-gleaning and herbivore squirrels showing the lowest values. This is logical because the moment arm of the superficial masseter is determined by the height of the mandibular ramus and both groups exhibit somewhat elongated mandibles with a low ramus. Consistently, the moment arm for the most ventral fibers of the temporalis also increases along CV2.

It is worth noting that the pattern expressed by the CVA is similar to that expressed by the PCA (see previous section and Fig. 3). PC2 expresses a decrease in the mechanical advantages of all mandibular muscles and places bark gleaners at one end of the axis and herbivore squirrels at the other, thus mimicking shape differences expressed by CV1 (see Table 2 and compare Figs. 3 and 4). PC1 reflects an increase in the mechanical advantage of the superficial masseter and the ventral fibers of the temporalis as described for CV2 (see Table 2 and compare Figs. 3 and 4). We may conclude therefore that functional demands fulfil a major role in the evolution of the squirrel mandible.

CV3 accounts for 19.0% of the variance and places all folivore flying squirrels at the negative end of the axis, and the granivore ground squirrels within the subfamilies Xerinae and Callosciurinae at the positive end (Fig. S4). The mandibles of granivore squirrels are very characteristic, showing a low and somewhat elongated shape with a shallow diastema and a forwards-directed lower incisor. Even though the incisor tip is not considered in our calculations, the orientation of the teeth can be inferred from its alveolus which is characteristically oblique to the mandibular corpus in such squirrels with forward-directed incisors. On the contrary, in many squirrels that feed on fruits or/and nuts the mandible is shorter and higher with vertically oriented incisors that tend to be robust. Certain squirrels that are predominantly folivores, such as *Petaurista* or *Pteromys*, take this trend even further and are characterized by high mandibles with a short corpus and a deep diastema. Some of these features (deep corpus and diastema) are also observed in bark-gleaning squirrels, which also have low values of CV3. The apophyses do not change markedly along CV3, although the angular process is displaced more dorsally in granivore ground squirrels.

Finally, CV4 accounts for an additional 9.5% of the variance but is difficult to interpret. Apparently it reflects the height of the mandibular corpus and the shape of the masseteric ridges (Fig. S4). To the positive side, the corpus becomes more robust and the upper and lower masseter ridge meet at a higher angle and more anteriorly than in mandibles that show negative values in this axis (Fig. S4). Extreme positive values are attained by squirrels which feed on hard nuts and fruits, such as *Rheithrosciurus* and *Protoxerus*. These squirrels exhibit short mandibles with a robust corpus and a high ramus. Their incisors are vertically oriented and powerful. At the other end of CV4 we find folivore and insectivore squirrels, characterized by lower and more slender mandibles.

The CVA discriminating modes of locomotion takes into account three different categories: terrestrial, arboreal/scansorial and gliding. In agreement with Michaux *et al*. [16] the results are very good, with 91.9% of correctly classified cases that decrease to 88.7% after cross-validation (see Table S4). Miss-classified specimens are mostly restricted to the terrestrial group which are sometimes classified as arboreal/scansorial. Terrestrial species usually possess elongated mandibles coupled with a forwards-directed incisor (Fig. S5). In contrast, arboreal/scansorial and gliding species have mandibles that tend to be higher with the incisor oriented more vertically (Fig. S5). The mandible of gliding squirrels is somewhat lower and more slender than that of arboreal/scansorial species. Obviously these shape differences between locomotory modes interact with those between dietary groups (see below).

In order to compare our results with previous studies of squirrel mandible shape we conducted separate CVAs using broad dietary preferences and locomotion types as defined by Michaux *et al*. [16]. The dietary categories distinguished by these authors recognize just three groups: plant-eater, plant-dominated omnivore and animal-dominated omnivore. The CVA correctly classifies 82.4% of the original cases while 78.8% of them are still correctly classified after cross-validation (see Table S5). The performance of this analysis is comparable to that of our CVA while using seven different dietary categories.

## Discussion

### Allometric trends

Our results indicate that allometry can only account for a small percent of shape variation as already reported by previous workers [21], [41], [47]–[49] so that scaling of the mandible and jaw muscles in squirrels does not deviate importantly from isometry. Swiderski and Zelditch [21] also showed that in the case of the Sciurinae isometry also extends to the mandibular lever arms, which were calculated in the same way as done in our work. Only slight deviations from isometry would occur at the extreme of the size range with dwarf squirrels, which appear to be associated with a shortening of the coronoid and angular processes coupled with a more elongated articular process and a forward shift in the position of the masseteric ridges [21], [41]. Velhagen & Roth [41] noted that the reduction of the coronoid process is most conspicuous in pigmy squirrels from the subfamily Callosciurinae, and propose an explanation in terms of space constraints: a large process for the insertion of a well-developed medial temporalis muscle would interfere with space requirements for the eye globe [22], [50]. On the other hand, Hautier *et al*. [22] pointed out that small-sized flying squirrels retain a long coronoid process, the shape of which does not differ much from that of their larger relatives. These authors relate the absence of allometry in the coronoid process to the retention of anteriorly positioned eyes, which are crucial for distance estimation in flying squirrels. By placing the eyes more anteriorly, interference with the medial masseter and the coronoid is avoided. According to these studies allometric trends for some individual subfamilies and tribes are expected to be different. This should be particularly true for the Pteromyini because dwarf flying squirrels present a different shape as compared to other dwarf squirrels within other tribes, such as *Nannosciurus*, *Sciurillus* or *Myosciurus* [20]. However, our analyses do not find significant differences in the slope nor intercept of the different squirrel subfamilies or tribes. Furthermore, small terrestrial Marmotini such as *Ammospermophilus* or *Tamias* show hook-shaped elongated coronoid processes, similar to those of minute flying squirrels, instead of the reduced ones that should be expected according to the functional hypothesis of Hautier *et al*. [20].

Even though allometry only explains a small part of the shape variation, our results indicate that the coronoid becomes reduced with decreasing body size and at the same time the articular process becomes elongated and the masseteric ridges are displaced more anteriorly, a pattern that has also been found by previous workers [20]–[21], [41], [47]. These trends, which are apparent in the dwarf squirrels *Nannosciurus*, *Sciurillus* and *Myosciurus*, can well be explained functionally (see below). In other small-sized squirrels some of these size-related shape changes can be detected, though they are not so evident. For example, the dwarf flying squirrel *Petaurillus* shows an articular process as long as that of the medium-sized *Hylopetes* or the giant flying squirrel *Petaurista*. On the other hand, the latter two species show a coronoid process which is more robust.

The differences between Hautier *et al*.'s study [22] and ours may result from the differences in method, since the former used the outline of the mandible [22], whereas we use a selection of anatomical points, including an interior one. Alternatively, the discrepancy may arise from differences in the species dataset, as Hautier *et al*. [22] included very few ground squirrels (Xerinae), which comprise true ‘giants’ such as *Marmota*, *Cynomys* or *Urocitellus*.

### Phylogeny versus function

Our results indicate that phylogeny fulfils an important role in determining mandible shape in squirrels, both in the whole family and in the main clades. In another geometric morphometrics study dealing with squirrels (Marmotini) a strong phylogenetic was found as well [48]. Also in other rodent groups, such as the Ctenohystrica, the importance of phylogeny has been demonstrated [51]. The pylogenetic structure in our dataset is clearly illustrated by the PCA, with many closely related taxa plotting close to one another in multivariate space (Figs. 3a, S1–S3). The clustering is stronger for ground squirrels (Marmotini and Xerini), flying squirrels (Pteromyini) and many Callosciurinae. This does not mean that functional explanations should be excluded. On the contrary, the first two principal components can be well interpreted in terms of the mechanical advantages of the main jaw muscles (Table 2).

**Figure 5:**
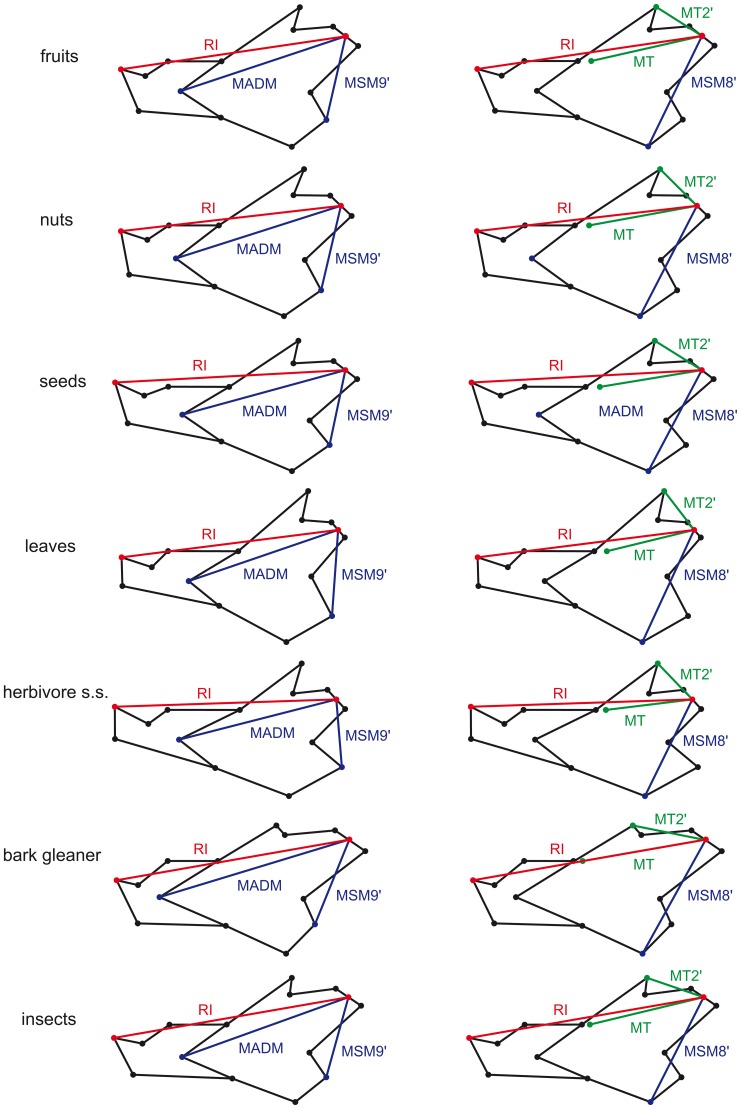
Muscle moment arms and resistance arm for the incisor for the mean shape of the main dietary groups. The numerical values for the mean of each group are given in Table 2. Values for each squirrel species are given in Table 1. See Figure 1 for the meaning of the acronyms.

The Xerinae provide an interesting example of how function has interfered with phylogeny. This subfamily includes three different tribes (Fig. 3b): Xerini (African ground squirrels), Protoxerini (African tree squirrels) and Marmotini (Holartic ground squirrels). Marmotini except for *Tamias* and *Sciurotamias* are predominantly herbivores, although an important proportion of other vegetable matter such as seeds are consumed as well (Fig. 3b, Table S1). *Sciurotamias* and *Tamias* are mostly granivores (Fig. 3b; Table S1). Interestingly, these two genera are the ones that diverged earlier from the Protoxerini than all the remaining Marmotini [18], [38]. Their body shape is also intermediate between ground and tree squirrels. The herbivore Marmotini plot very close to one another in the multivariate space (Figs. 3a, S2) reflecting a close phylogenetic relationship. However, at the same time the pattern is functional, since they all adapted to a similar diet (Figs. 3a, S2) with mandibles characterized by a lower mechanical advantage of all muscles compared to other squirrels (Fig. 5; Tables 1–2). Xerini plot very near to the Marmotini although they are phylogenetically closer to the Protoxerini(Fig. 3b). Apparently, this is the case because their dietary preferences are to be the primary agent determining mandible shape: both Xerini and Marmotini have evolved low and elongated mandibles with low mechanical advantages for all mandibular muscles.

On the other hand, the mandible shape of the Protoxerini is markedly different from that of the Marmotini and Xerini with fruits and nuts dominating the diet (Fig. 3b; Table S1). Also their mandible shape also reflects an adaptation to process specific dietary items. For example, the mandibles of *Protoxerus* and *Heliosciurus*, large-sized tree squirrels that feed on hard nuts and fruits, recalls those similarly-sized squirrels such as *Ratufa* (Ratufinae) or *Rheithrosciurus* (Sciurinae), which also feed on fruits and nuts (Figs. 3, S1–S2). All these squirrels have powerful jaw muscles that can provide the strong incisor bite that is required for opening hard-shelled nuts and fruits (see below). Interestingly, the only diet-related exception within the Protoxerini, the specialized bark gleaner *Myosciurus*, has a mandible shape which is very similar to that of other bark gleaners such as *Nannosciurus* (Callosciurinae) or *Sciurillus* (Sciurillinae) (Figs. 3, S1–S2). These squirrels are characterized by robust mandibles with a reduced coronoid, a projecting articular process and an anteriorly placed masseteric ridge (Fig. 5, Tables 1–2). Such a configuration increases the mechanical advantage of many mandibular muscles, most markedly the anterior deep masseter and the temporalis, providing a powerful incisor bite and aiding in a rapid retraction of the mandible (see below).

This discussion can be further extended towards the Sciurinae and Callosciurinae. The Sciurinae have specialized in a limited range of diets. The flying squirrels of the *Pteromys*-*Petaurista* clade are predominantly folivores and plot close to each other in the scatter of the first two PCs (Fig. 3a). The woolly flying squirrel *Eupetaurus*, on the other hand, has specialized in consuming pine needles [46]. Consistently, its mandible shape diverges from that of more ‘standard’ flying squirrels, approaching the morphology of the herbivore Marmotini characterized by elongated mandibular corpuses, narrow articular processes and posteriorly-placed masseteric ridges (Figs. 3a, S1). At the same time, however, *Eupetaurus* retains the high ramus that is typical for flying squirrels and lacks the elongated angular apophysis as seen in the Marmotini. The possession of these synplesiomorphies may explain why our CVA wrongly assigns *Eupetaurus* to the folivore group (Table S3).

The Callosciurinae have adapted to a broader spectrum of diets than other squirrel clades. Even though many Callosciurinae, such as *Funambulus*, *Callosciurus* and *Lariscus*, are mostly frugivores, their diets also include an important percent of other vegetal and even animal matter. This clade also includes bark-gleaners (*Nannosciurus*, *Exilisciurus*), insectivores (*Rhinosciurus*) and granivores (*Menetes*). As can be seen in Fig. 3b, closely related genera do not always have the similar dietary preferences (Fig. 3b, Table S1). This point explains why our tests fail to find a phylogenetic signal for this clade.

Previous studies have suggested that in rodents in general the mode of locomotion [16] or substrate preference [51] may be more important in determining mandible shape than diet. Particularly Michaux *et al*. [16] outlined the role of the mode of locomotion in shaping the mandible of squirrels. Although our CVA using this parameter as grouping variable gives slightly better results than that based on dietary preferences (Table S4) we believe that the shapes for the three locomotor groups (gliding, arboreal/scansorial and terrestrial) indeed reflect dietary preferences. E.g. terrestrial squirrels show low and elongated mandibles with a long diastema, a forwards-pointing incisor, a reduced coronoid process and a somewhat elongated and dorsally deflected angular process (Fig. S5). This morphology recalls that of granivores and herbivores (Fig. 5) which make up most of the terrestrial squirrels. Arboreal/scansorial squirrels have robust and high mandibles, with the masseteric ridge placed anteriorly and all mandibular processes well developed (Fig. S5). This morphology recalls that of squirrels feeding on fruits and nuts (Fig. 5) that define the majority of tree squirrels. Finally, the mandible of gliding squirrels equals that of folivore squirrels (Fig. 5) differing from that of arboreal/scansorial squirrels by a longer coronoid process, a broader angular process and a more reduced articular process. This is not surprising, since all folivore squirrels are also flying squirrels.

To sum up, our results indicate that diet is probably the primary external factor determining squirrel mandible shape. This is not inconsistent with the strong phylogenetic structure in the data, as closely related taxa tend to share dietary preferences. The Callosciurinae represent an exception containing widely different dietary specializations evolved in relatively short time, causing measures of phylogenetic structure to be low.

### Mandibular mechanics of the main dietary groups

The main axes in both the PCA and the CV (Figs. 3a, 4) reflect changes in the mechanical efficiencies of the jaw muscles associated with different moment arms (Fig. 5; Table 2). As already noted by Swiderski and Zelditch [21], even if they are small, the differences in the arm lengths of the masticatory muscles are functionally important.

The most characteristic mandible shapes are perhaps shown by the bark gleaners. This group includes squirrels belonging to three different subfamilies: the Sciurillinae *Sciurillus*, the Xerinae *Myosciurus* and the Callosciurinae *Nannosciurus* and *Exilisciurus*. All forms are distinguished by very short mandibles with reduced coronoids, elongated articular processes and masseteric ridges placed anteriorly (Fig. 5). Such morphology results in high mechanical advantages for all muscles (Table 2), particularly for the anterior deep masseter. The more anterior insertion of the deep masseter increases the strength of incisor bite [40], [52]–[53]. Additionally this muscle may aid in retraction of the mandible [47]. Bark gleaners feed by grasping and vigorously yanking fragments of bark [47], [54]. This requires forceful gnawing with the anterior dentition in order to mechanically damage the trees and elicit tree exudates [55]. Increasing the mechanical advantage of the anterior deep masseter is therefore crucial for forceful incision [40], [52]–[53] aiding in rapidly dislodging bark chips. Since all the bark gleaners are pigmy squirrels one may expect that their particular mandible morphology may be somehow associated with body-size scaling. However, mandibles of other small-sized squirrels, such as *Petaurillus* or *Ammospermophilus* (see Figs. S1–S2), are clearly different. Therefore, the characteristic morphology of bark gleaners truly reflects their adaptation to a unique mode of feeding. A different, unanswered question is why all the bark gleaners are dwarf squirrels. We tentatively suggest that this may be due to interspecific competition with larger squirrels. Bite force is positively correlated with body size [21], [56], so larger squirrels can exert greater forces to open hard-shelled fruits and nuts than smaller ones. Competition with larger squirrels and other larger herbivorous mammals would have forced dwarf squirrels to specialize on a dietary activity such as bark gleaning. This would have occurred independently in the three different subfamilies that inhabit tropical and subtropical forests.

The mandibles of insectivores and granivore squirrels are very close to one another in shape, and the mechanical advantages of the muscles are similar (Fig. 5; Table 2). The mandibles are low and elongated, with a long shallow diastema and a well-developed articular process, particularly in the insectivores. The coronoid and angular apophysis are somewhat more reduced in the latter group. The mandibles of both groups are characterized by an overall low mechanical advantages for all muscles, preventing a strong incisor bite as in squirrels that feed on harder items such as nuts. The highly specialized, exclusively insectivorous *Rhinosciurus* takes this trend towards the extreme. Its mandible is shrew-like: low and elongated with a long articular process and a reduced angular one. These squirrels patrol the forest floor of Malaysia and Indonesia feeding on insects, earthworms and other invertebrates [43]. The elongated shrew-like rostrum shows other adaptations to insectivory as well, including a long protrusible tongue. The mechanical advantages for most muscles are low, particularly for the superficial and anterior deep masseter (Table 1). Because the moment arms depend on the depth of the mandible only a weak incisor bite is possible, which is in accordance with its characteristically reduced upper incisors. On the other hand, the mechanical advantage for the most dorsal fibers of the temporalis is higher than in the other insectivore squirrels (*Dremomys*, *Tamiops* and *Microsciurus*; Table 1). These fibers would provide a rapid retraction of the mandible which in turn may aid this squirrel in capturing its prey. A reduction of the mandibular muscles coupled with a degeneration of the incisors and cheek teeth has been observed in other insectivorous rodents as well [57].

The Marmotini tribe includes the majority of ground squirrels that feed on grasses. They are characterized by a low and elongated mandibles showing a reduced mechanical advantage for all muscles (Fig. 5; Table 2). The angular process is broad and long, while the articular one is short and narrow. The coronoid process is not reduced. Characteristically, the cheek tooth row is elongated as compared to other squirrels. It has been suggested for the chipmunk *Tamias* that the presence of well-developed cheek pouches, a synapomorphy within the Marmotini, may have determined its mandible shape [47]. Here we observe that other ground squirrels feeding on seeds show a similar mandible shape (Figs. 3, S2), even if they have very reduced cheek pouches (*Sciurotamias*) or not cheek pouches at all (in the Xerini). We therefore assume that the mandible shape of herbivore squirrels simply reflects the fact that they feed on items that do not require a powerful incisor bite. A recent finite element analysis of extant rodent skulls by Cox *et al* [58] has shown that rodents that feed on fruits and nuts (such as *Sciurus*) are more efficient with their incisor bite than those that feed on grass (such as *Cavia*). The latter are more efficient in chewing and grinding using the molars [58]. This is consistent with our results. At the same time the elongated cheek tooth row may reflect their greater efficiency grinding the plant material. In the CVA, *Eupetaurus*, which feeds on pine needles [46], is placed in the herbivore *sensu stricto* category. While its mandibular corpus is remarkably similar to that of the herbivore Marmotini, its ramus is conspicuously higher showing a shorter angular process recalling that of other flying squirrels (Fig. S1). The higher ramus of *Eupetaurus* provides an overall higher mechanical advantage for the superficial masseter and will allow a stronger incisor bite with regard to herbivore squirrels (Table 1). The ramus shape may therefore represent a synplesiomorphy shared with other flying squirrels or alternatively may reflect that the consumption of pine needles requires a strong incisor bite.

The mandibles of folivore squirrels such as *Trogopterus* or *Belomys* share some features with those of the herbivore ones. In both groups the angular processes are broader and longer than in the remaining squirrels, whereas the articular process is low and narrow (Fig. 5). The corpus is low and elongated, but the ramus is not as low as in herbivores and granivores, but as high as in frugivores and nut eaters. The mechanical advantages of the individual muscles of herbivore and folivore squirrels are roughly comparable, but the latter show a conspicuous mechanical advantage for the superficial masseter which is comparable to that of squirrels that feed on nuts and fruits (Table 2). This similarity can be explained considering the secondary food items consumed. Whereas herbivores may consume also seeds, insects, tubers or roots, folivores such as *Belomys* and specially *Petaurista* secondarily or seasonally consume fruits and nuts [43]. The consumption of these items requires a more powerful incisor bite, which may explain the retention of a higher ramus.

Mandible shapes and associated mechanical advantages are very similar in fruit-eaters and nut eaters (Table 2). Both show high and relatively short mandibles (Fig. 5) and differ from all other squirrels by higher mechanical advantages for all muscles (Table 2), particularly for the superficial masseter. Harder fruits and particularly nuts are protected by hard shells making them mechanically resistant against fracture [59]–[60]. Accordingly many mammals have evolved morphological adaptations for forceful biting with their incisors and/or cheek teeth [61]–[62]. Additionally, in *Rheithrosciurus*, *Protoxerus* and *Ratufa*, the insertion of the anterior deep masseter is displaced forward, providing a mechanical advantage comparable to that of the bark gleaners (Table 1). This adaptation is observed in other hard-object-feeding mammals as well (including bats, primates and other rodents). They often exhibit more anteriorly positioned muscles and/or posterior migration of the cheek teeth, a configuration that will improve not only the mechanical advantage during incisor bite but also during mastication [62]–[65]. The forceful biting of these squirrels is associated with the possession of stout incisors, especially in *Rheithrosciurus* [40].

## Conclusions and Implications for Squirrel Evolution

Our study clearly shows that the mandible shape of squirrels reflect their dietary specialization, and can be used to predict diet with significant reliability. This conclusion fits the results of a recent study across the rodent order [57], which shows that cranial shape is also a good proxy for feeding habits. On the other hand, it allows reliable inferences on the diet of fossil species as has been done for the earliest squirrel *Douglassciurus jeffersoni* in this work and for extinct fossorial beavers by Samuels [57].

The dominant mandibular shape in extant squirrels is characterized by a robust corpus, a high ramus and all processes well developed. It is found in nut-eaters and frugivores for which it provides a high mechanical advantage of all mandibular muscles. The folivores, which all belong to the flying squirrel clade, show similar mandibles except for a more elongated corpus resulting in lower mechanical advantages. The mandibles of the granivores, insectivores and herbivores *sensu stricto* are low and elongated and have low mechanical advantages (particularly for the herbivores and certain insectivores). The lower mechanical advantages for all these groups reflect the fact that they feed on items that do not require a powerful incisor bite. Finally, the mandible of bark gleaners is very characteristic. All the bark-gleaning genera are pigmy squirrels that show a short mandible with a robust corpus and a markedly elongated articular process, whereas the other mandibular apophyses are reduced. The masseteric ridge is placed in a more anterior position, providing a high mechanical advantage for the anterior deep masseter. At the same time, the elongated articular increases the mechanical advantage of the temporalis. Such morphology allows a forceful incisor bite, which is crucial for efficiently dislodging bark chips.

The prediction of seven dietary categories based on CVA represents an important refinement with regard to earlier studies [16]. Even though mandible shape also reflects the dominant modes of locomotion, the relationship seems to be indirect, with shape rather controlled by the underlying diets than by ways of locomotion or feeding habitats themselves. Size is probably less important than previously thought [22], [41]. For example, the mandible shapes of pygmy squirrels largely reflect a dietary specialization on bark gleaning.

While clearly being dependent on diet, mandible shape in squirrels contains a strong phylogenetic signal as well. The important role of phylogeny is explained by the retention by most squirrels of the the ancestral nut- and fruit-dominated diet, and by the relatively slow radiation of early-branching groups dominated by other dietary types (e.g. herbivory in the Marmotini or folivory in the *Pteromys*-*Petaurista* clade). Nevertheless, interesting “cross-over” developments have occurred: for example Protoxerini (Xerinae) mandibles are more similar to those of large-sized tree squirrels of the subfamilies Ratufinae and Sciurinae than to the ones of other tribes from their own subfamily (Xerini and Marmotini). These two tribes represent ground squirrels with similarly-shaped low mandibles, even though the latter are more closely related to the Protoxerini than to the Xerini. In some cases, squirrels were able to adapt drastically and rapidly. This is well evidenced by the specialized group of bark gleaners, whose few members belong to Sciurillinae, Xerinae and Callosciurinae that have converged towards similar mandible shapes. Another example represents *Rhinosciurus*, which has acquired an insectivorous lifestyle within the Callosciurinae, thereby showing convergent evolution with other specialized insectivore mammals.

Summarizing our findings and referring to the title of the paper we may conclude that conservatism is a major feature in squirrel evolution. However, the occurrence of highly specialized forms demonstrates that conservatism is not an intrinsic feature of squirrels. Apparently, the early squirrel model was successful from its origin and remained relatively unchanged.

With regard to these earliest forms, it is interesting to note that Thorington & Darrow [40] speculated that sciuromorphous mandibles could have arisen as an adaptation for feeding on hard fruits, and that only afterwards particular clades would have adapted to other diets. Our analyses challenge this hypothesis, as *Douglassciurus* is classified amongst the nut and seed eaters, showing that the oldest squirrels (Late Eocene, 36 Ma) already fed on these items even though they were protrogomorphous. A minority of the squirrels have deviated from this ancestral diet, resulting in an unbalanced and hererogeneous occupation of the multivariate morphospace (Fig. 3a, Fig. 4). The Xerinae apparently deviated earlier than other groups developing more elongated mandibles. The oldest undoubted members of this clade are known from the Late Oligocene (between 26 and 24 Ma) of Europe (*Heteroxerus*) [66]–[67] and North America (*Nototamias*) [68]–[69]. The Protoxerini, which likewise evolved in Africa, would have secondarily returned to a diet based on fruits and nuts in combination with an arboreal lifestyle. Consistently, the postcranial skeleton of the oldest known Protoxerini, *Kubwaxerus pattersoni* from the Late Miocene (about 8–6 Ma) of Kenya [70], shows that it still was terrestrial. On the other hand, its massive skull and mandible with extraordinarily deep lower incisors resembles that of squirrels that feed on hard nuts such as *Ratufa*, *Rheithrosciurus* or *Protoxerus*. As explained before, the African pigmy squirrel (*Myosciurus pumilio*) even adapted to bark gleaning with a mandible shape converging to that of other minute squirrels with similar dietary habits [40].

The highest morphological diversity is found within the Callosciurinae, with an estimated Early Miocene divergence date of 21 Ma [18] and with the oldest fossils (Pakistan) dating back to the Middle Miocene [71]. The Callosciurinae include highly specialized squirrels (such as the only exclusively insectivorous genus and most of the bark gleaners. This is probably related to the exploitation of the diverse and continuously available food resources of the tropical and subtropical forests of Southeastern Asia, where this subfamily is particularly diverse. MacKinnon [72] and Payne [73] showed that as many as 16 different squirrel species (mostly Callosciurinae) coexisted in their study area in north-central Malaysia. These species were foraging at different heights of the canopy, during different daytimes and were exploiting different food resources. Furthermore, many Callosciurinae species are restricted to the Sunda Shelf islands, where variations in sea level may have played an important role facilitating dispersal during low stands and allopatric speciation on islands during high stands [18].

Also in the tropical forests of Africa squirrel diversity is high especially when compared to the temperate forests of Europe and North America. As in Southeastern Asia, African diversity may also reflect ecological niche partitioning [74]. By contrast, squirrel diversity is lower in the tropical forests of South America, with only two to four species coexisting in a locality [54], [75]. This, in turn, may be related to the relatively recent dispersal of squirrels into South America, which, with the exception of *Sciurillus*, probably took place after the formation of the Panama Isthmus during the Pliocene three-four million years ago.

## Supporting Information

Figure S1 **Principal Component Analysis (PCA) of mandible shape, Sciurinae phylogeny and dietary preferences.** Size-corrected PCA of covariance matrix among species means for the Sciurinae with a projection of the phylogenetic tree in the PC1-PC2 plot. The black mandible outlines represent shape changes with respect to consensus configuration (grey outline).
(TIF)

Figure S2 **Principal Component Analysis (PCA) of mandible shape, Xerinae phylogeny and dietary preferences.** Size-corrected PCA of covariance matrix among species means for the Xerinae with a projection of the phylogenetic tree in the PC1-PC2 plot. The black mandible outlines represent shape changes with respect to consensus configuration (grey outline).
(TIF)

Figure S3 **Principal Component Analysis (PCA) of mandible shape, Callosciurinae phylogeny and dietary preferences.** Size-corrected PCA of covariance matrix among species means for the Sciurinae with a projection of the phylogenetic tree in the PC1-PC2 plot. The black mandible outlines represent shape changes with respect to consensus configuration (grey outline).
(TIF)

Figure S4 **Figure 4** **. Canonical Variates Analysis (CVA) of squirrel mandible shape using dietary preferences as grouping variable.** Plot of CV3 against CV4. Summary of classification results: see Table 3. Results for each particular case: see Table S3.
(TIF)

Figure S5 **Mean shapes of the main locomotor groups.** Grey outline represents consensus shape.
(TIF)

Table S1 **List of squirrel species included in this study.** For each species the number of specimens (n), geographical distribution, main habitat and diet, together with supplementary references for the data sources are given.
(DOCX)

Table S2 **Probability of equality between all pairs of dietary groups in the Canonical Variates Analysis.** Probability of equality (*p*) as well as Procrustes distances (Pdist) between all possible pairs of groups are given. Note that the tests find significant differences between all groups. For further details see main text, Figures 4, S4, Table 3 and Table S3.
(DOCX)

Table S3 **Results of Canonical Variates Analysis of all specimens using dietary preferences as grouping variable.** *n* refers to specimen number, whereas ‘group’ indicates the original dietary group. Probabilities of group membership (*p*) are given for predicted group and the next most probable group together with cross validated probabilities and discriminant scores. Miss-classified cases in bold. For a summary of classification results: see Table 3. For further details: see main text and Figures 4, S4 and Table S2.
(DOCX)

Table S4 **Summary of classification results of Canonical Variates Analysis (CVA) of squirrel mandible shape using the dietary categories of Michaux** ***et al*** **.** [**16**] **as grouping variable.**
(DOCX)

Table S5 **Summary of classification results of Canonical Variates Analysis (CVA) of squirrel mandible shape using the locomotion categories of Michaux** ***et al*** **.** [16] as grouping variable. The fossil *Douglassciurus jeffersoni* was assigned to the arboreal/scansorial group with *p* = 0.816.
(DOCX)
