# Supplementary figures and images for: Conservatism and Adaptability during Squirrel Radiation: What Is Mandible Shape Telling Us?
Source: PLoS One. 2013 Apr 4;8(4):e61298. doi: 10.1371/journal.pone.0061298 (PMC3617180; doi:10.1371/journal.pone.0061298)

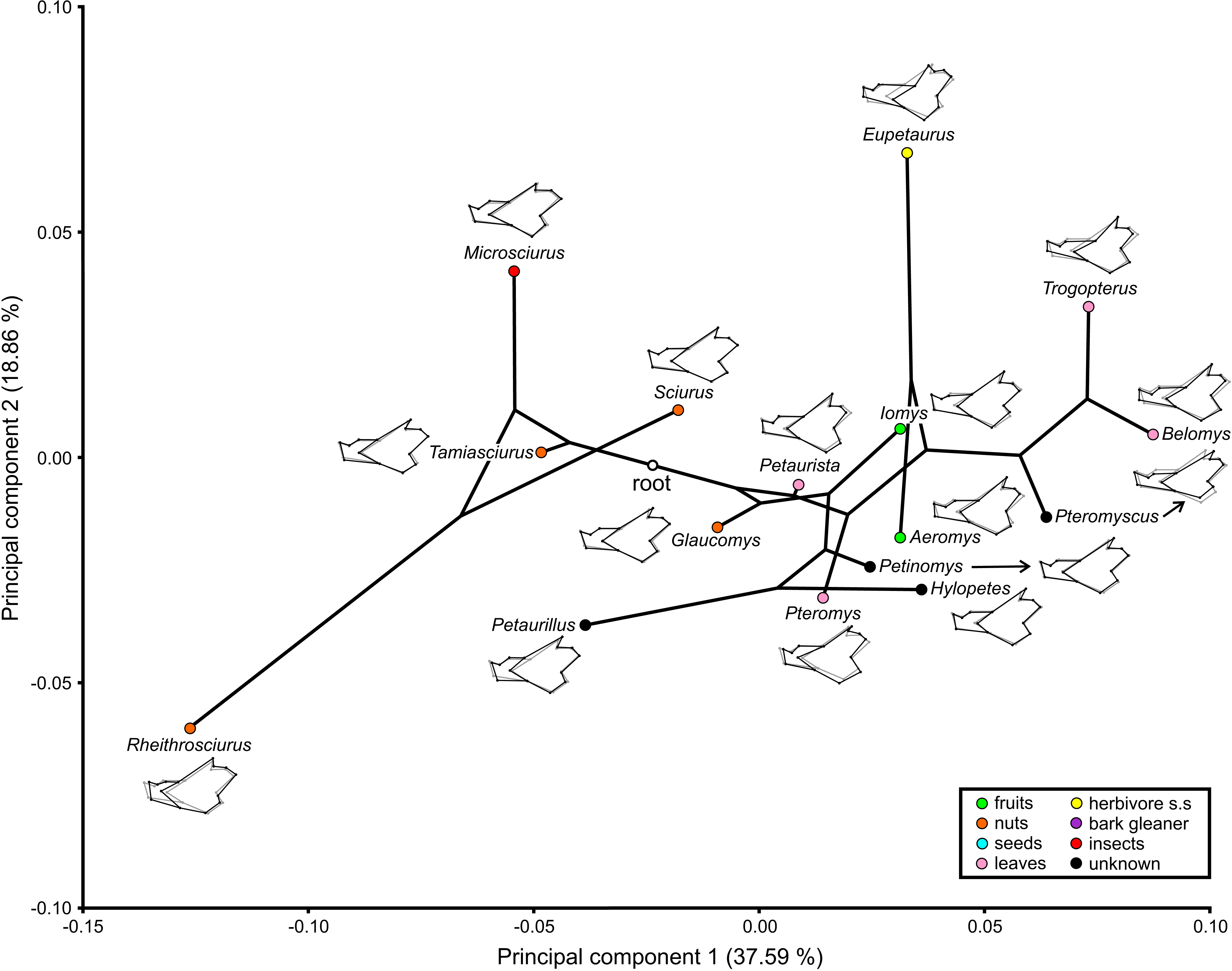

Supplement: Figure S1 — Principal Component Analysis (PCA) of mandible shape, Sciurinae phylogeny and dietary preferences. Size-corrected PCA of covariance matrix among species means for the Sciurinae with a projection of the phylogenetic tree in the PC1-PC2 plot. The black mandible outlines represent shape changes with respect to consensus configuration (grey outline). (TIF) [file pone.0061298.s001.tif]

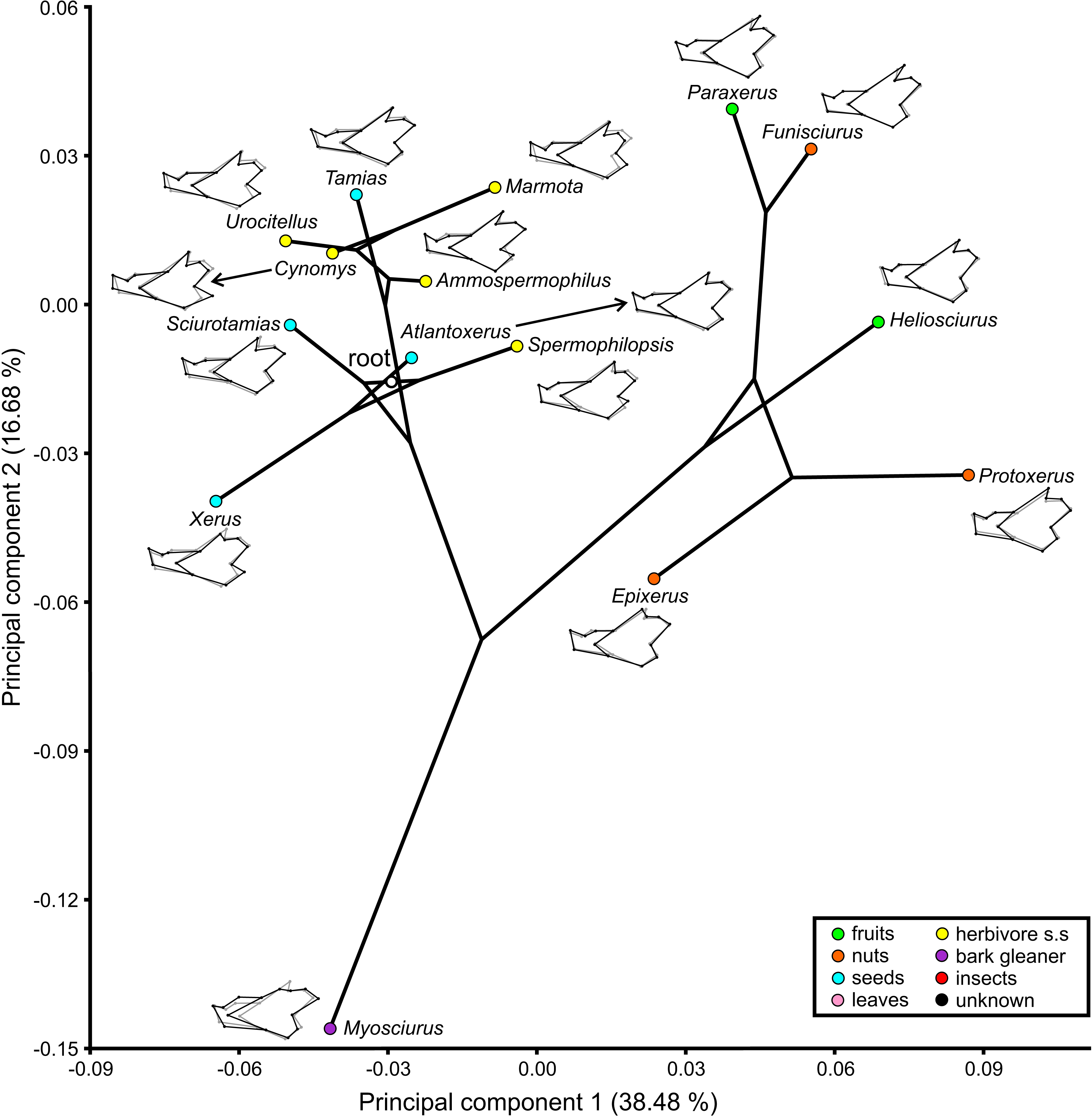

Supplement: Figure S2 — Principal Component Analysis (PCA) of mandible shape, Xerinae phylogeny and dietary preferences. Size-corrected PCA of covariance matrix among species means for the Xerinae with a projection of the phylogenetic tree in the PC1-PC2 plot. The black mandible outlines represent shape changes with respect to consensus configuration (grey outline). (TIF) [file pone.0061298.s002.tif]

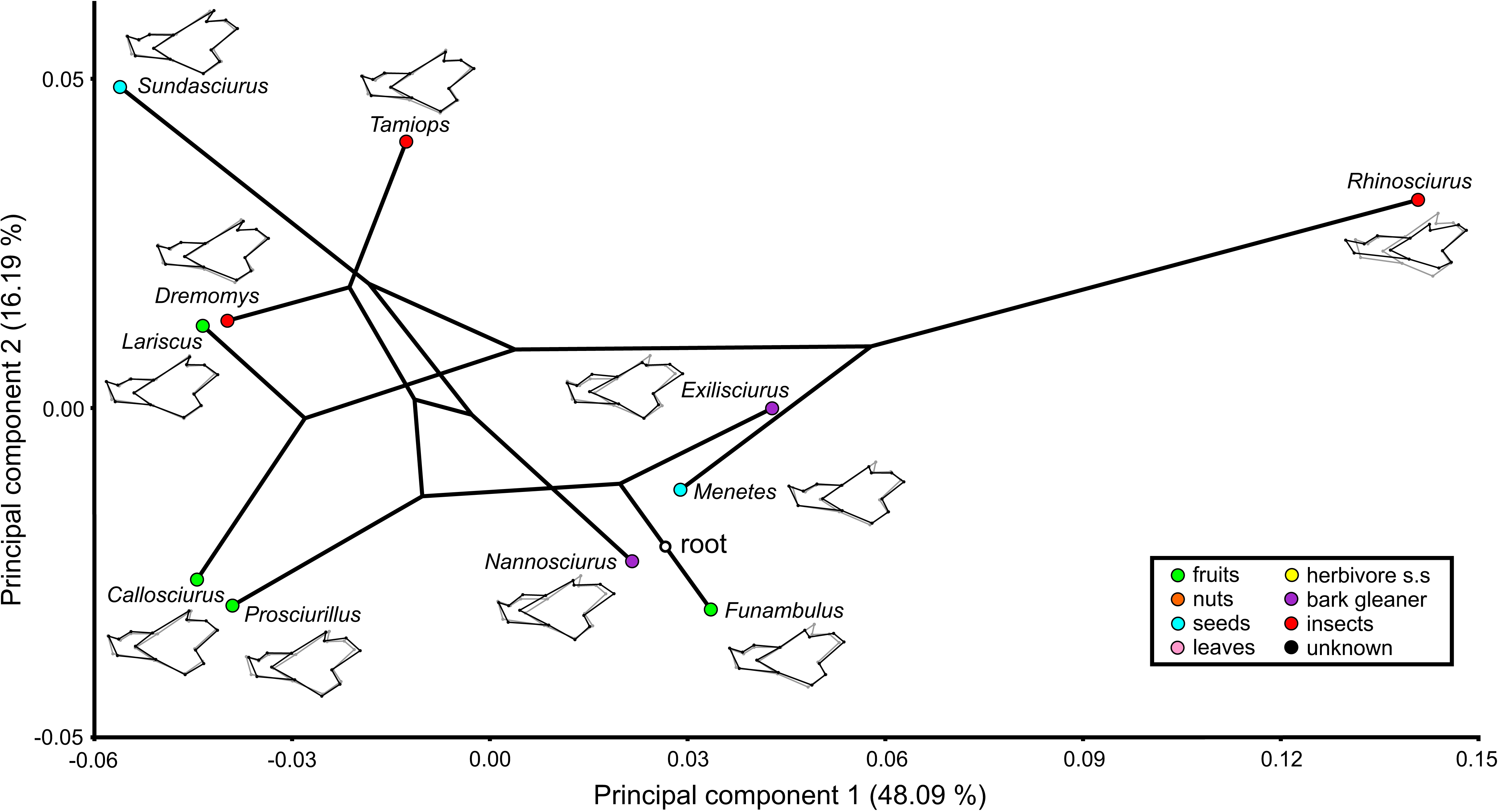

Supplement: Figure S3 — Principal Component Analysis (PCA) of mandible shape, Callosciurinae phylogeny and dietary preferences. Size-corrected PCA of covariance matrix among species means for the Sciurinae with a projection of the phylogenetic tree in the PC1-PC2 plot. The black mandible outlines represent shape changes with respect to consensus configuration (grey outline). (TIF) [file pone.0061298.s003.tif]

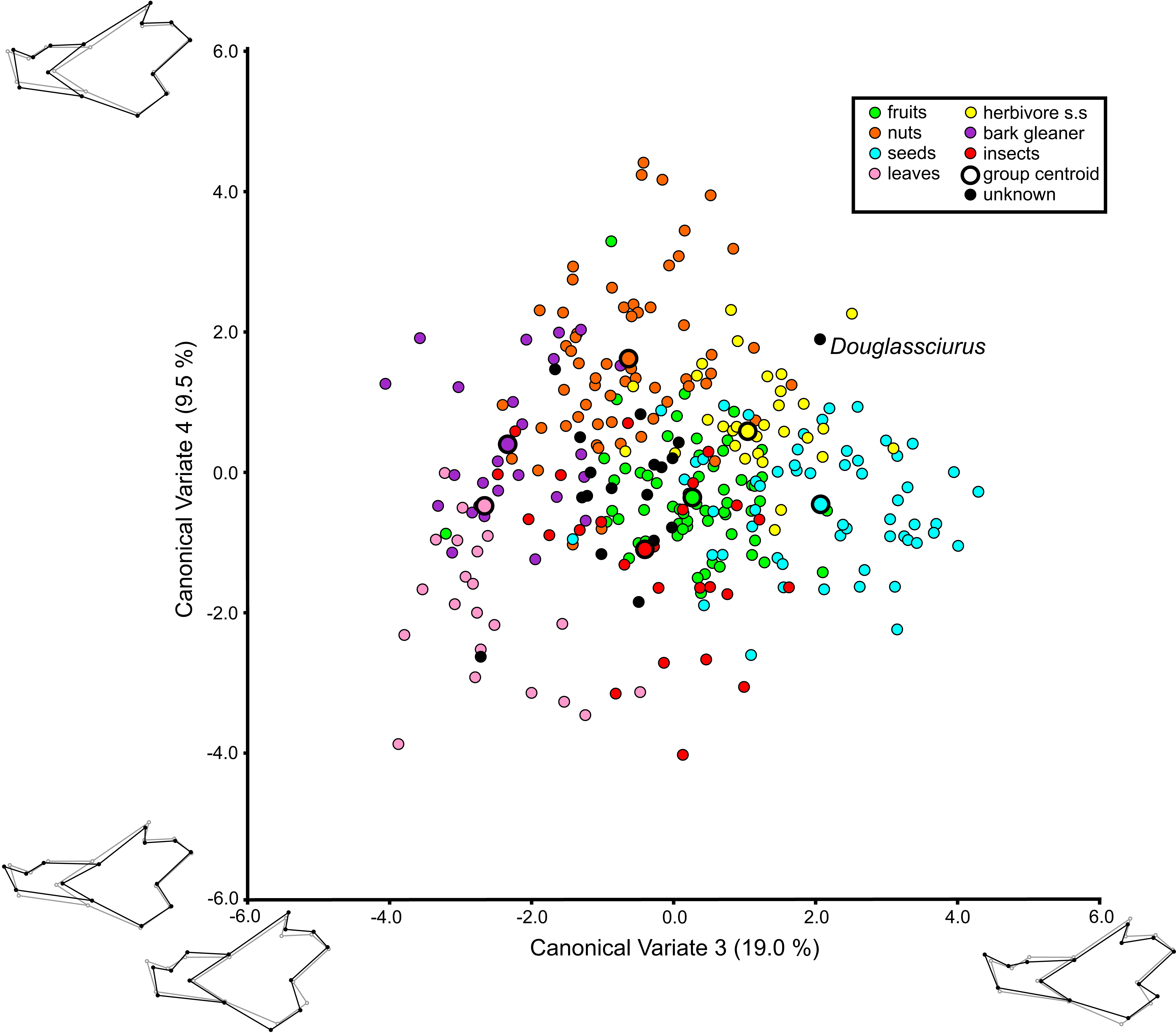

Supplement: Figure S4 — Figure 4 . Canonical Variates Analysis (CVA) of squirrel mandible shape using dietary preferences as grouping variable. Plot of CV3 against CV4. Summary of classification results: see Table 3. Results for each particular case: see Table S3. (TIF) [file pone.0061298.s004.tif]

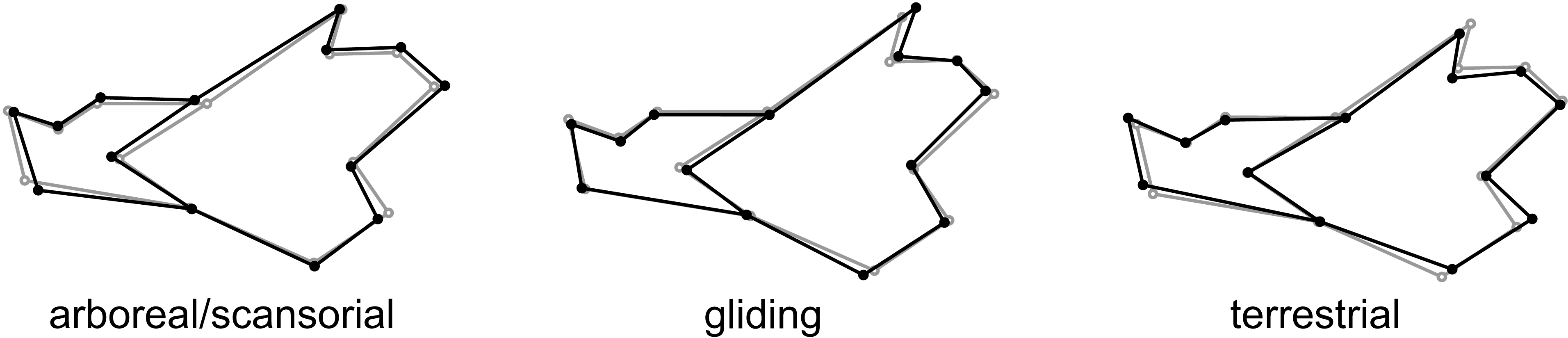

Supplement: Figure S5 — Mean shapes of the main locomotor groups. Grey outline represents consensus shape. (TIF) [file pone.0061298.s005.tif]
