# Supplementary material for: Conservatism and Adaptability during Squirrel Radiation: What Is Mandible Shape Telling Us?
Source: PLoS One. 2013 Apr 4;8(4):e61298. doi: 10.1371/journal.pone.0061298 (PMC3617180; doi:10.1371/journal.pone.0061298)
Supplement: Table S1 — List of squirrel species included in this study. For each species the number of specimens (n), geographical distribution, main habitat and diet, together with supplementary references for the data sources are given. (DOCX) [file pone.0061298.s006.docx]

**Table S1. List of squirrel species included in this study.** For each species the number of specimens (n), geographical distribution, habitat and dietary preferences and locomotion, together with the references for the data sources are given. Thorington *et al.* [S1] is used as a general reference for habitat and feeding preferences for extant squirrels. Dietary preferences are listed from the most important to the least important items.

| Subfamily | Tribe | Species | n | Distribution | Habitat preferences | Dietary preferences | References |
| --- | --- | --- | --- | --- | --- | --- | --- |
| ? | ? | *Douglassciurus jeffersoni* (fossil) | 1 | North America | - | - | S2-S3 |
| Sciurillinae |  | *Sciurillus pusillus* | 7 | N South America | tropical rainforest | bark gleaner | S4-S7 |
| Ratufinae |  | *Ratufa bicolor* | 10 | SE Asia and Indonesia | tropical rainforest, tropical deciduous forest | nuts, fruits, leaves, flowers, sap, bark | S8-S10 |
| Sciurinae | Sciurini | *Microsciurus flaviventer* | 10 | Amazon Basin | tropical rainforest | insects, sap and other exudates obtained gnawing wood | S4,S11 |
|  |  | *Rheithrosciurus macrotis* | 2 | Borneo | tropical rainforest | fruits, nuts, thick-shelled seeds, insects | S12-S13 |
|  |  | *Sciurus vulgaris* | 10 | Europe, N and central Asia | coniferous forest, deciduous forest, Mediterranean forest | conifer nuts and seeds, fruits, buds, flowers, shoots, herbs | S14-S16 |
|  |  | *Tamiasciurus hudsonicus* | 10 | North America | coniferous forest, deciduous forest | conifer nuts and seeds, seeds of deciduous trees, flowers, buds, fresh fruits, tree sap, bark, insects | S17-S20 |
|  | Pteromyini | *Aeromys tephromelas* | 1 | Malay Peninsula and Indonesia | tropical rainforest | fruits, nuts, seeds and other plant material | S21 |
|  |  | *Belomys pearsonii* | 4 | SE Asia | warm-temperate forest, deciduous forest, mixed deciduous-coniferous forest | leaves, fruits, nuts, seeds and other plant material | S22 |
|  |  | *Eupetaurus cinereus* | 1 | Himalaya (Pakistan) | rocky areas (highland), dry conifer forest | pine needles | S23 |
|  |  | *Glaucomys volans* | 7 | E North America and certain areas of Central America | deciduous forest, mixed deciduous-coniferous forest | nuts, seeds, fruits, fungi, lichens, moss, bark, animal matter | S24-S27; |
|  |  | *Hylopetes lepidus* | 7 | Indonesia (Java and Bangka) | tropical rainforest | unknown | S28 |
|  |  | *Iomys horsfieldii* | 3 | Indonesia | tropical rainforest (prefers less dense areas), plantations | fruits | S29 |
|  |  | *Petaurillus kinlochii* | 1 | Indonesia (Malaysia) | tropical rainforest (prefers less dense areas), plantations | Unknown | S30 |
|  |  | *Petaurista petaurista* | 10 | Himalaya, SE Asia, Indonesia | tropical rainforest (prefers less dense areas), warm-temperate forest, coniferous forest, deciduous forest, plantations | leaves, seeds, fruits, flowers, buds, bark and lichens | S31-S34 |
|  |  | *Petinomys genibarbis* | 10 | Malay Peninsula and Indonesia | tropical rainforest (secondary forests), plantations | unknown | S35 |
|  |  | *Pteromys volans* | 1 | N Eurasia | coniferous forest, mixed deciduous-coniferous forest | leaves, pine needles, buds, bark, lichens and fruits | S36-S38 |
|  |  | *Pteromyscus pulverulentus* | 1 | Malay Peninsula and Indonesia | tropical rainforest | unknown | S39 |
|  |  | *Trogopterus xantiphes* | 6 | central China | mixed deciduous-conifer forest, rochy areas | leaves | S40 |
| Callosciurinae |  | *Callosciurus erythraeus* | 10 | SE continental Asia | tropical rainforest (highland), warm-temperate forest, mixed deciduous-coniferous forest | fruits, insects, flowers and other vegetable matter, bird eggs | S41 |
|  |  | *Dremomys rufigenis* | 5 | SE continental Asia | tropical rainforest (highland) | insects, fruits, nuts, seeds and other plant material | S42 |
|  |  | *Exilisciurus exilis* | 6 | Borneo | tropical rainforest (highland) | bark gleaner, insects | S9 |
|  |  | *Funambulus palmarum* | 8 | India and Sri Lanka | tropical deciduous forest, tropical rainforest, plantations | fruits, nuts, buds, young shoots, bark, nectar, insects | S43 |
|  |  | *Lariscus insignis* | 10 | Malay Peninsula and Indonesia | tropical rainforest, secondary forests | fruits, insects, buds of *Rafflesia* flowers* | S9, S44-S45 |
|  |  | *Menetes berdmorei* | 10 | Malay Peninsula | tropical rainforest, plantations | seeds, leaves, fruits | S46-S47 |
|  |  | *Nannosciurus melanotis* | 10 | Indonesia | tropical rainforest | bark gleaner | S48-S49 |
|  |  | *Prosciurillus leucomus* | 8 | Sulawesi | tropical rainforest, tropical deciduous forest, coastal areas | fruits, insects | S50 |
|  |  | *Rhinosciurus laticaudatus* | 7 | Malay Peninsula and Indonesia | tropical rainforest (lowland and highland) | ants and termites, other invertebrates | S13, S51 |
|  |  | *Sundasciurus altitudinis* | 10 | Sumatra | tropical rainforest (highland) | seeds | S52 |
|  |  | *Tamiops mcclellandii* | 5 | SE Asia | tropical rainforest, warm-temperate forests, plantations | insects, fruits and other vegetable matter | S53 |
| Xerinae | Xerini | *Atlantoxerus getulus* | 10 | NW Africa | shrubland, open woodland, temperate grassland, desert, rocky areas | seeds , fruits, snails | S54 |
|  |  | *Spermophilopsis leptodactylus* | 1 | loess steppes SE Caspian Sea | sandy desert | seeds, fruits, bulbs, insects, roots and other vegetation | - |
|  |  | *Xerus erythropus* | 10 | central Africa | open woodlands, grasslands, swamp forest and mangrove, secondary forest | seeds, fruits, leaves, flowers, other vegetable matter, insects and small vertebrates | S55-S57 |
|  | Protoxerini | *Epixerus ebii* | 3 | coastal W Africa | tropical rainforest (lowland and highland), tropical deciduous forest | nuts, fruits, insects | S58 |
|  |  | *Funisciurus congicus* | 10 | W-central Africa | tropical deciduous forest, coastal palm groves | seeds, fruits, nuts, stems, shoots and occasionally insects | S59-S60 |
|  |  | *Heliosciurus gambianus* | 10 | tropical central Africa | woodland savannah, tropical deciduous forest, seasonally flooded grassland, rainforest, plantations | fruits, seeds, nuts, insects, acacia pods, small vertebrates | S56-S57,S61 |
|  |  | *Myosciurus pumilio* | 2 | W-central Africa | tropical rainforest | bark gleaner | S58 |
|  |  | *Paraxerus ochraceus* | 10 | Kenya, Tanzania | Savannah, tropical deciduous forest, riparian tropical forests | fruits, seeds, buds, flowers, roots, buds, acacia gum, small animals | S57,S62 |
|  |  | *Protoxerus stangeri* | 10 | W and Central Africa | tropical rainforest, tropical deciduous forest, secondary forest, plantations | nuts, fruits, seeds and other plant material, arthropods | S58,S63 |
|  | Marmotini | *Ammospermophilus leucurus* | 2 | W USA | arid plains, desert, rocky areas | stems and roots, seeds, insects | S64 |
|  |  | *Cynomys ludovicianus* | 7 | Central USA | arid grasslands | grasses and forbs | S65 |
|  |  | *Marmota marmota* | 10 | central and Alpine mountains of Europe | alpine meadow, steppe | grasses and forbs, seeds, fruits | S66 |
|  |  | *Sciurotamias davidianus* | 6 | mountains of China | rocky areas | seeds, nuts | S53,S67-S69 |
|  |  | *Tamias striatus* | 10 | E North America | deciduous forest | large seeds (acorns), nuts, insects, nesting birds and other small animals, fungi | S70-S72 |
|  |  | *Urocitellus undulatus* | 10 | central Asian steppe | steppe, semidesert, savannah | green shoots, leaves, flowers, seeds, opportunistically may consume insects and small animals | S53, S73 |

*Other *Lariscus species*, such as *L.* *obscurus*, feed primarily on fruits and seeds with insects being also a major staple in their diets [45]. *L. insignis* is assumed to be primarily frugivore in our analyses

**Supplementary references**

S1. Thorington RWJr, Koprowski JL, Steele MA, Whatton JF (2012) Squirrels of the World. Baltimore: The Johns Hopkins University Press. 459 p.

S2. Emry RJ, Thorington RWJr (1982) Descriptive and comparative osteology of the oldest fossil squirrel, *Protosciurus* (Rodentia: Sciuridae). Smithson Contrib Paleobiol 47: 1-35.

S3. Emry RJ, Korth WW (1996) The Chadronian squirrel “*Sciurus*” *jeffersoni* Douglass, 1901: a new generic name, new material, and its bearing on the early evolution of Sciuridae (Rodentia). J Vert Pal 16: 775-780.

S4. Emmons LH, Feer F (1980) Neotropical Rainforest Mammals. Chicago: Chicago University Press. 396 p.

S5. Amori G, Koprowski J, Roth L (2008) *Sciurillus pusillus*. In: IUCN 2012. IUCN Red List of Threatened Species. Version 2012.2. Available: www.iucnredlist.org/ details/19997/0

S6. Anthony HE, Tate GHH (1935) Notes on South American Mammalia: No. 1, *Sciurillus*. Am Mus Novit 780: 1-13.

S7. Heymann EW, Knogge C (1997) Field observations on the Neotropical pigmy squirrel, *Sciurillus pusillus* (Rodentia: Sciuridae) in Peruvian Amazonia. Ecotropica 3: 67-69.

S8. Payne JB (1979) Synecology of Malayan tree squirrels with special reference to the genus *Ratufa*. PhD thesis, University of Cambridge, Cambridge.

S9. Payne JB (1980) Competitors. In: Chivers DJ, editor. Malayan Forest Primates: Ten Years’ Study in Tropical Rain Forest. New York: Plenum Press. pp. 261-277.

S10. Moore JC, Tate GHH (1965) A study of the diurnal squirrels, Sciurinae, of the Indian and Indochinese subregions. Fieldiana Zoology 48: 1-372.

S11. Buitrón-Jurado G, Tobar M (2007) Posible asociación de la ardilla enana *Microsciurus flaviventer*(Rodentia: Sciuridae) y bandadas mixtas de aves en la Amazonia ecuatoriana. Mastozool Neotrop 14: 235-240.

S12. Duckworth JW, Meijaard E (2010) *Rheithrosciurus macrotis*. In: IUCN 2012. IUCN Red List of Threatened Species. Version 2012.2. Available: www.iucnredlist.org/ details/19474/0

S13. Payne J, Francis CM (1985) A Field Guide to the Mammals of Borneo. Kuala Lumpur: Sabah Society. 332 p.

S14. Lurz PWW, Gurnell J, Magris L (2005) *Sciurus vulgaris*. Mammalian Species 769. 1-10.

S15. Shar S, Lkhagvasuren D, Bertolino S, Henttonen H, Kryštufec B, Meinig H (2008) *Sciurus vulgaris*. In: IUCN 2012. IUCN Red List of Threatened Species. Version 2012.2. Available: www.iucnredlist.org/ details/20025/0

S16. Wauters LA, Vermeulen M, Van Dongen S, Bertolino S, Molinari A, Tosi G, Matthysen E (2007) Effects of spatio-temporal variation in food supply on red squirrel *Sciurus vulgaris* body size and body mass and its consequences for some fitness components. Ecography 30: 51-65.

S17. Banfield AWF (1974) The Mammals of Canada. University of Toronto Press, Toronto. 438 p.

S18. Steele MA (1998) *Tamiasciurus hudsonicus*. Mammalian Species 586: 1-9.

S19. Layne JN (1954) The biology of the red squirrel, *Tamiasciurus hudsonicus loquax* (Bangs), in central New York. Ecol Monogr 24: 227-268.

S20. Smith CC (1970) The coevolution of pine squirrels (*Tamiasciurus*) and conifers. Ecol Monogr 40: 349-371.

S21. Aplin K, Lunde D, Duckworth JW, Lee B, Tizard RJ (2008) *Aeromys tephromelas*. In: IUCN Red List of Threatened Species. Version 2012.2. Available: http://www.iucnredlist.org/details/556/0

S22. Duckworth JW, Molur S (2008) *Belomys pearsonii*. In: IUCN 2012. IUCN Red List of Threatened Species. Version 2012.1. www.iucnredlist.org/details/2756/0

S23. Zahler P, Khan M (2003) Evidence for dietary specialization on pine needles by the woolly flying squirrel (*Eupetaurus cinereus*). J Mammal 84: 480-486.

S24. Linzey AV, Hammerson G, (2008) *Glaucomys volans*. In: IUCN 2012. IUCN Red List of Threatened Species. Version 2012.2. Available: http://www.iucnredlist.org/details/9240/0

S25. Dolan PG, Carter DC (1977) *Glaucomys volans*. Mammalian Species 78: 1-6.

S26. Harlow RF, Doyle AT (1990) Food habits of southern flying squirrels (*Glaucomys volans*) collected from red-cockaded woodpecker (*Picoides borealis*) colonies in South Carolina. Am Midl Nat 124: 187-191.

S27. Sawyer SL, Rose RK (1985) Homing in and ecology of the southern flying squirrel *Glaucomys volans* in southeastern Virginia. Am Midl Nat 113: 238-244.

S28. Duckworth JW, Hedges S (2008) *Hylopetes lepidus*. In: IUCN Red List of Threatened Species. Version 2012.2. Available: http://www.iucnredlist.org/details/10603/0

S29. Aplin K, Lunde D (2008) *Iomys horsfieldii*. In: IUCN Red List of Threatened Species. Version 2012.2. Available: http://www.iucnredlist.org/details/10845/0

S30. Francis C, Duckworth JW, (2008) *Petaurillus kinlochii*. In: IUCN Red List of Threatened Species. Version 2012.2. Available: http://www.iucnredlist.org/details/16716/0

S31. Lambert F (1990) Some notes on fig-eating by arboreal mammals in Malaysia. Primates 31: 453-458.

S32. Lee PF, Progulske DR, Lin YS (1986) Ecological studies on two sympatric *Petaurista* species in Taiwan. Bulletin of the Institute of Zoology, Academia Sinica 25: 113-124.

S33. Shafique CM, Barkati S, Oshida T, Ando M (2006). Comparison of the diets between two sympatric flying squirrels in northern Pakistan. J Mammal 87: 784-789.

S34. Walston J, Duckworth JW, Sarker SU, Molur S (2008) *Petaurista petaurista*. In: IUCN Red List of Threatened Species. Version 2012.2. http://www.iucnredlist.org/details/16723/0

S35. Francis C, Gumal M, (2008) *Petinomys genibarbis*. In: IUCN Red List of Threatened Species. Version 2012.2. Available: http://www.iucnredlist.org/details/16735/0

S36. Airapetyants AE, Fokin IM (2003) Biology of European flying squirrel *Pteromys volans* L. (Rodentia: Pteromyidae) in the North-Wst of Russia. Russ J Theriol 2. 105-113.

S37. Mönkkönen M, Reunanen P, Nikula A, Inkeröinen J, Forsman J (1997) Landscape characteristics associated with the occurrence of the flying squirrel *Pteromys volans* in old-growth forests of northern Finland. Ecography 20: 634-642.

S38. Shar S, Lkhagvasuren D, Henttonen H, Maran T, Hanski I (2008) *Pteromys volans*. In: IUCN Red List of Threatened Species. Version 2012.2. Available: http://www.iucnredlist.org/details/18702/0

S39. Muul I, Liat LB (1971) New locality records for some mammals of West Malaysia. J Mammal 52: 430-437.

S40. Wang F (1985) Preliminary study of the ecology of *Trogopterus xantiphes*. Acta Theriologica Sinica 5: 103-110.

S41. Setoguchi M (1990) Food habits of red-bellied tree squirrels on a small island in Japan. J Mammal 71: 570-578.

S42. Endo H, Kimura J, Oshida T, Stafford BJ, Rerkamnuaychoke W, Nishida T, Sasaki M, Hayashida A, Hayashi Y (2003) Geographical variation of skull morphology and its fuctional significances in the red-cheeked squirrel. Journal of Veterinary Medical Science 65: 1179-1183.

S43. Nameer PO, Molur S (2008) *Funambulus palmarum*. In: IUCN 2012. IUCN Red List of Threatened Species. Version 2012.2. Available: www.iucnredlist.org/details/8701/0

S44. Hedges S, Duckworth JW, Lee B, Tizard RJ (2008) *Lariscus insignis*. In: IUCN Red List of Threatened Species. Version 2012.2. Available: http://www.iucnredlist.org/details/11305/0

S45. Whitten JEJ (1981) Ecological separation of three diurnal squirrels in tropical rainforest on Siberut Island, Indonesia. Journal of Zoology 193: 405-420.

S46. Cao VS, Pham DT, Tran VM, Nguyen MT, Kuznetsov GV, Kuljukina NM (1986) Écologie des rongeurs de forêt tropicale du Vietnam. Mammalia 50: 323-328.

S47. Walston J, Duckworth JW (2008) *Menetes berdmorei*. In: IUCN Red List of Threatened Species. Version 2012.2. Available: http://www.iucnredlist.org/details/13144/0

S48. Heaney LR (1985) Systematics of Oriental Pigmy Squirrels of the Genera *Exilisciurus* and *Nannosciurus* (Mammalia: Sciuridae). Miscellaneous Publication 170. Ann Arbor: University of Michigan, Museum of Zoology. 58 p.

S49. Francis C, Gumal M, Han KH, (2008) *Nannosciurus melanotis*. In: IUCN Red List of Threatened Species. Version 2012.2. Avilable: http://www.iucnredlist.org/details/14325/0

S50. Musser GG, Durden LA, Holden ME, Light JE (2010) Systematic of Sulawesi squirrels (Rodentia, Sciuridae) with descriptions of new species of associated sucking lice (Insecta, Anoplura), and phylogenetic and zoogeographic assessments of sciurid lice. Bull Am Mus Nat Hist 339: 1-260.

S51. Lekagul B, McNeely JA (1977) Mammals of Thailand. Bangkok: Sahakarnbhat. 758 p.

S52. den Tex RJ, Thorington RWJr, Maldonado JE, Leonard JA (2010) Speciation dynamics in the SE Asian tropics: Putting a time perspective on the phylogeny and biogeography of Sundaland tree squirrels, *Sundasciurus*. Mol Phylogenet Evol 55: 711-720.

S53. Smith AT, Xie Y, eds (2008) A Guide to the Mammals of China. Princeton, NJ: Princeton University Press. 576 p.

S54. López-Darias M, Nogales M (2008) Effects of the invasive Barbary ground squirrel (*Atlantoxerus getulus*) on seed dispersal systems of insular xeric environments. J Arid Environ 72:926.

S55. Herron MD, Waterman JM (2004) *Xerus erythropus*. Mammalian Species 748: 1-4.

S56. Delany MJ, Happold DCD (1979) Ecology of African Mammals. New York: Longman.

S57. Kingdon J (1974) East African Mammals: An Atlas of Evolution in Africa. Hares and Rodents. Vol. 2, pt. B. New York: Academic Press. 371 p.

S58. Emmons LH (1980) Ecology and resource partitioning among nine species of African rain forest squirrels. Ecol Monogr 50: 31-54.

S59. Viljoen S (1978) Notes on the western striped squirrel, *Funisciurus congicus congicus* (Kuhl, 1820). Madoqua 11: 119-128.

S60. Viljoen S (1997) Striped tree squirrel. In: Mills MGL, Hes L, editors. The Complete Book of Southern African Mammals. Cape Town: Struik.

S61. Meester J, Setzer HW (1977) The Mammals of Africa: An Identification Manual*.* Washington DC: Smithsonian Institution Press 483 p.

S62. Grubb P (2008) *Paraxerus ochraceus*. In: IUCN 2011. IUCN Red List of Threatened Species. Version 2012.2. Available: www.iucnredlist.org/details/16209/0

S63. Gautier-Hion A, Emmons LH, Dubost G (1980) A comparison of the diets of three major groups of primary consumers of Gabon (primates, squirrels and ruminants). Oecologia 45: 182-189.

S64. Belk MC, Smith HD (1991) *Ammospermophilus leucurus*. Mammalian Species 368: 1-8.

S65. Hoogland JL (1996). *Cynomys ludovicianus*. Mammalian Species 535: 1-10.

S66. Mann CS, Macchi E, Janeau G (1993) Alpine marmot (*Marmota marmota* L.) Ibex Mongraph 1: 17-30.

S67. Lu J, Zhang Z (2007) Hoarding of walnuts by David’s rock squirrels (*Sciurotamias davidianus*) within enclosure. Acta Theriologica Sinica 292: 209-214.

S68. Wang W, Zhang HM, Zhang ZB (2007) Effects of predation risk on cultivated walnut (*Juglans regia*) seeds hoarding behavior by David’s rock squirrel (*Sciurotamias davidianus*) in enclosure. Acta Theriologica Sinica 27: 358-364.

S69. Zhang ZB, Xiao ZS, Li HJ (2005) Impact of small rodents on tree seeds in temperate and subtropical forests, China. In: Forget P-M, Lambert JE, Hulme PE, Vander Wall SB, editors. Seed Fate: Predation, Dispersal and Seedling Establishment. Cambridge: CAB International Publishing. pp. 269-282.

S70. Bowers MA (1995) Use of space and habitats by the eastern chipmunk, *Tamias striatus*. J Mammal 87. 60-66.

S71. Godin AJ (1977) Wild Mammals of New England. Baltimore: The Johns Hopkins University Press. 123 p.

S72. Snyder DP (1982) *Tamias striatus.* Mammalian Species 168: 1-8.

S73. Ricankova V, Fric Z, Chlachula J, Stastna P, Faltynkova A, Zemek F (2006) Habitat requirements of the long-tailed ground squirrel (*Spermophilus undulatus*) in southern Altai. Journal of Zoology 270: 1-8.
