# Supplementary material for: Conservatism and Adaptability during Squirrel Radiation: What Is Mandible Shape Telling Us?
Source: PLoS One. 2013 Apr 4;8(4):e61298. doi: 10.1371/journal.pone.0061298 (PMC3617180; doi:10.1371/journal.pone.0061298)
Supplement: Table S2 — Probability of equality between all pairs of dietary groups in the Canonical Variates Analysis. Probability of equality (p) as well as Procrustes distances (Pdist) between all possible pairs of groups are given. Note that the tests find significant differences between all groups. For further details see main text, Figures 4, S4, Table 3 and Table S3. (DOCX) [file pone.0061298.s007.docx]

**Table S2. Probability of equality between all pairs of dietary groups in the Canonical Variates Analysis.** Probability of equality (*p*) as well as Procrustes distances (Pdist) between all possible pairs of groups are given. Note that the tests find significant differences between all groups. For further details see main text, Figures 4, S4, Table 3 and Table S3.

|  | Fruits | | Nuts | | Seeds | | Leaves | | Herbivore s.s. | | Bark gleaner | | Insects | |
| --- | --- | --- | --- | --- | --- | --- | --- | --- | --- | --- | --- | --- | --- | --- |
|  | **Pdist** | ***p*** | **Pdist** | ***p*** | **Pdist** | ***p*** | **Pdist** | ***p*** | **Pdist** | ***p*** | **Pdist** | ***p*** | **Pdist** | ***p*** |
| Fruits | - | *-* | 0.036 | *<0.0001* | 0.071 | *<0.0001* | 0.099 | *<0.0001* | 0.135 | *<0.0001* | 0.121 | *<0.0001* | 0.075 | *<0.0001* |
| Nuts | 0.036 | *<0.0001* | - | *-* |  |  | 0.093 | *<0.0001* | 0.138 | *<0.0001* | 0.134 | *<0.0001* | 0.097 | *<0.0001* |
| Seeds | 0.071 | *<0.0001* | 0.089 | *<0.0001* | - | *-* | 0.103 | *<0.0001* | 0.090 | *<0.0001* | 0.134 | *<0.0001* | 0.057 | *0.0002* |
| Leaves | 0.100 | *<0.0001* | 0.093 | *<0.0001* | 0.103 | *<0.0001* | - | *-* | 0.100 | *<0.0001* | 0.193 | *<0.0001* | 0.130 | *<0.0001* |
| Herbivore s.s. | 0.135 | *<0.0001* | 0.138 | *<0.0001* | 0.090 | *<0.0001* | 0.100 | *<0.0001* | - | *-* | 0.200 | *<0.0001* | 0.133 | *<0.0001* |
| Bark gleaner | 0.121 | *<0.0001* | 0.134 | *<0.0001* | 0.134 | *<0.0001* | 0.193 | *<0.0001* | 0.200 | *<0.0001* | - | - | 0.096 | *<0.0001* |
| Insects | 0.075 | *<0.0001* | 0.097 | *<0.0001* | 0.057 | *0.0002* | 0.130 | *<0.0001* | 0.133 | *<0.0001* | 0.096 | *<0.0001* | - | - |
