# Supplementary material for: Conservatism and Adaptability during Squirrel Radiation: What Is Mandible Shape Telling Us?
Source: PLoS One. 2013 Apr 4;8(4):e61298. doi: 10.1371/journal.pone.0061298 (PMC3617180; doi:10.1371/journal.pone.0061298)
Supplement: Table S3 — Results of Canonical Variates Analysis of all specimens using dietary preferences as grouping variable. n refers to specimen number, whereas ‘group’ indicates the original dietary group. Probabilities of group membership (p) are given for predicted group and the next most probable group together with cross validated probabilities and discriminant scores. Miss-classified cases in bold. For a summary of classification results: see Table 3. For further details: see main text and Figures 4, S4 and Table S2. (DOCX) [file pone.0061298.s008.docx]

**Table S3.** **Results of Canonical Variates Analysis of all specimens using dietary preferences as grouping variable.** *n* refers to specimen number, whereas ‘group’ indicates the original dietary group. Probabilities of group membership (*p*) are given for predicted group and the next most probable group together with cross validated probabilities and discriminant scores. Miss-classified cases in bold. For a summary of classification results: see Table 3. For further details: see main text and Figures 4, S4 and Table S2.

| Species | n | Group | Major group | | Second major group | | Cross-validation | | Discriminant scores | | | | | |
| --- | --- | --- | --- | --- | --- | --- | --- | --- | --- | --- | --- | --- | --- | --- |
|  |  |  | **Predicted group** | **p** | **Predicted group** | **p** | **Predicted group** | **p** | **CV1** | **CV2** | **CV3** | **CV4** | **CV5** | **CV6** |
| *Aeromys tephromelas* | - | fruits | **leaves** | 0.997 | nuts | 0.003 | **leaves** | 1.000 | -2.925 | 2.218 | -3.216 | -0.879 | -0.857 | 0.249 |
| *Ammospermophilus leucurus* | 1 | herbivore ss | **seeds** | 0.996 | nuts | 0.001 | **seeds** | 0.997 | -0.278 | -1.345 | 2.104 | 0.614 | -1.520 | 0.546 |
| *Ammospermophilus leucurus* | 2 | herbivore ss | **seeds** | 0.713 | herbivore ss | 0.170 | **seeds** | 0.862 | -1.593 | -1.066 | 1.475 | 0.926 | -0.880 | 0.604 |
| *Atlantoxerus getulus* | 1 | seeds | seeds | 0.532 | nuts | 0.380 | **nuts** | 0.669 | -1.039 | -0.389 | 0.708 | 0.911 | -0.670 | -1.690 |
| *Atlantoxerus getulus* | 2 | seeds | seeds | 0.900 | fruits | 0.096 | seeds | 0.849 | 0.206 | -0.537 | 1.537 | -1.310 | -0.172 | -0.950 |
| *Atlantoxerus getulus* | 3 | seeds | seeds | 0.953 | fruits | 0.029 | seeds | 0.934 | -0.572 | -0.334 | 1.109 | -0.795 | -1.052 | 0.116 |
| *Atlantoxerus getulus* | 4 | seeds | seeds | 0.953 | fruits | 0.045 | seeds | 0.936 | -0.655 | 0.618 | 1.461 | -1.238 | -1.017 | -1.647 |
| *Atlantoxerus getulus* | 5 | seeds | seeds | 0.958 | fruits | 0.042 | seeds | 0.935 | 0.931 | 1.106 | 2.374 | -0.789 | -0.915 | -1.651 |
| *Atlantoxerus getulus* | 6 | seeds | seeds | 0.999 | fruits | 0.000 | seeds | 0.999 | 0.421 | -0.314 | 3.372 | 0.380 | -1.504 | 0.131 |
| *Atlantoxerus getulus* | 7 | seeds | seeds | 0.885 | fruits | 0.092 | seeds | 0.796 | 0.122 | 0.681 | 1.150 | -0.163 | -1.175 | -0.854 |
| *Atlantoxerus getulus* | 8 | seeds | seeds | 0.980 | fruits | 0.020 | seeds | 0.969 | 1.102 | -0.427 | 3.150 | 0.208 | -0.050 | -2.087 |
| *Atlantoxerus getulus* | 9 | seeds | seeds | 0.988 | fruits | 0.007 | seeds | 0.981 | -0.032 | 0.452 | 2.093 | 0.718 | -1.736 | -1.581 |
| *Atlantoxerus getulus* | 10 | seeds | seeds | 0.994 | fruits | 0.006 | seeds | 0.992 | 0.353 | 0.500 | 3.158 | -0.409 | -0.545 | 0.138 |
| *Belomys pearsonii* | 1 | leaves | leaves | 1.000 | nuts | 0.000 | leaves | 1.000 | -4.686 | 1.088 | -2.817 | -2.912 | -1.345 | -0.710 |
| *Belomys pearsonii* | 2 | leaves | leaves | 1.000 | herbivore ss | 0.000 | leaves | 1.000 | -4.753 | -0.255 | -2.956 | -1.478 | -0.511 | 1.014 |
| *Belomys pearsonii* | 3 | leaves | leaves | 1.000 | nuts | 0.000 | leaves | 1.000 | -5.018 | 1.246 | -1.587 | -2.150 | -1.222 | 0.354 |
| *Belomys pearsonii* | 4 | leaves | leaves | 1.000 | nuts | 0.000 | leaves | 1.000 | -4.576 | 0.126 | -2.792 | -1.996 | -0.745 | -0.802 |
| *Callosciurus erythraeus* | 1 | fruits | fruits | 0.960 | nuts | 0.019 | fruits | 0.951 | 1.114 | 0.679 | 0.320 | 0.443 | 1.501 | -0.106 |
| *Callosciurus erythraeus* | 2 | fruits | fruits | 0.987 | seeds | 0.011 | fruits | 0.982 | 1.729 | 1.626 | 0.644 | -1.357 | 1.296 | -0.841 |
| *Callosciurus erythraeus* | 3 | fruits | fruits | 0.982 | insects | 0.017 | fruits | 0.973 | 0.671 | 1.008 | 0.540 | -1.300 | 3.385 | 0.645 |
| *Callosciurus erythraeus* | 4 | fruits | fruits | 0.896 | seeds | 0.102 | fruits | 0.832 | 2.168 | 0.138 | 1.108 | -1.190 | 0.771 | -1.978 |
| *Callosciurus erythraeus* | 5 | fruits | fruits | 0.941 | nuts | 0.040 | fruits | 0.921 | 0.543 | 1.165 | -0.874 | -0.578 | 1.026 | -0.378 |
| *Callosciurus erythraeus* | 6 | fruits | fruits | 0.985 | seeds | 0.012 | fruits | 0.981 | 1.428 | 0.902 | 1.088 | -0.560 | 2.088 | -0.555 |
| *Callosciurus erythraeus* | 7 | fruits | fruits | 0.942 | seeds | 0.030 | fruits | 0.904 | 2.027 | 1.253 | 0.442 | -0.573 | 0.559 | -0.014 |
| *Callosciurus erythraeus* | 8 | fruits | fruits | 0.976 | insects | 0.015 | fruits | 0.963 | 0.608 | 1.381 | 0.049 | -0.904 | 1.867 | 0.236 |
| *Callosciurus erythraeus* | 9 | fruits | fruits | 0.920 | nuts | 0.048 | fruits | 0.890 | 0.768 | 0.686 | -0.475 | -0.031 | 0.966 | -0.216 |
| *Callosciurus erythraeus* | 10 | fruits | fruits | 0.958 | insects | 0.037 | fruits | 0.940 | 1.616 | 0.449 | -0.637 | -1.248 | 0.933 | -0.822 |
| *Cynomys ludovicianus* | 1 | herbivore ss | herbivore ss | 1.000 | seeds | 0.000 | herbivore ss | 1.000 | -4.580 | -3.008 | 2.518 | 2.213 | 0.946 | -0.524 |
| *Cynomys ludovicianus* | 2 | herbivore ss | herbivore ss | 1.000 | nuts | 0.000 | herbivore ss | 1.000 | -5.034 | -4.260 | 0.893 | 1.821 | 0.777 | 1.009 |
| *Cynomys ludovicianus* | 3 | herbivore ss | herbivore ss | 1.000 | nuts | 0.000 | herbivore ss | 1.000 | -5.289 | -3.346 | 1.530 | 1.359 | -0.978 | 0.836 |
| *Cynomys ludovicianus* | 4 | herbivore ss | herbivore ss | 1.000 | seeds | 0.000 | herbivore ss | 1.000 | -4.343 | -4.131 | 1.321 | 1.332 | 0.725 | 0.209 |
| *Cynomys ludovicianus* | 5 | herbivore ss | herbivore ss | 1.000 | seeds | 0.000 | herbivore ss | 1.000 | -4.156 | -3.325 | 1.213 | 0.233 | 0.029 | -0.745 |
| *Cynomys ludovicianus* | 6 | herbivore ss | herbivore ss | 1.000 | leaves | 0.000 | herbivore ss | 1.000 | -4.925 | -6.100 | -0.587 | 1.202 | 0.318 | 2.037 |
| *Cynomys ludovicianus* | 7 | herbivore ss | herbivore ss | 1.000 | leaves | 0.000 | herbivore ss | 1.000 | -4.821 | -2.911 | 0.887 | 0.609 | 0.541 | 0.614 |
| *Dremomys rufigenis* | 1 | insects | insects | 0.944 | seeds | 0.037 | insects | 0.845 | 1.448 | 0.544 | 0.270 | -0.170 | -1.370 | 2.926 |
| *Dremomys rufigenis* | 2 | insects | **fruits** | 0.663 | insects | 0.278 | **fruits** | 0.798 | 1.485 | 0.819 | 0.130 | -0.559 | 0.251 | 0.917 |
| *Dremomys rufigenis* | 3 | insects | **fruits** | 0.936 | insects | 0.026 | **fruits** | 0.952 | 0.742 | 0.868 | 0.482 | 0.259 | 1.857 | 0.747 |
| *Dremomys rufigenis* | 4 | insects | **fruits** | 0.558 | insects | 0.429 | **fruits** | 0.781 | 2.404 | 0.941 | 0.504 | -1.658 | 1.064 | 1.221 |
| *Dremomys rufigenis* | 5 | insects | insects | 0.442 | fruits | 0.398 | **fruits** | 0.526 | 1.771 | 0.821 | 1.212 | -0.690 | 0.442 | 1.775 |
| *Epixerus ebii* | 1 | nuts | nuts | 0.614 | seeds | 0.316 | **seeds** | 0.561 | 0.637 | 2.730 | 1.662 | 1.209 | -1.104 | 1.413 |
| *Epixerus ebii* | 2 | nuts | nuts | 0.999 | seeds | 0.001 | nuts | 0.997 | -1.045 | 0.573 | -1.548 | 1.139 | -3.096 | 0.438 |
| *Epixerus ebii* | 3 | nuts | nuts | 0.981 | fruits | 0.014 | nuts | 0.939 | -0.059 | 1.894 | -0.567 | 1.327 | -1.702 | -1.374 |
| *Eupetaurus cinereus* | 1 | herbivore ss | **leaves** | 0.999 | insects | 0.001 | **leaves** | 1.000 | -1.918 | 0.238 | -2.815 | -3.775 | -0.517 | 1.194 |
| *Exilisiciurus exilis* | 1 | bark gleaner | bark gleaner | 0.952 | insects | 0.048 | bark gleaner | 0.837 | 2.790 | -2.216 | -2.688 | -0.629 | -0.919 | 1.205 |
| *Exilisiciurus exilis* | 2 | bark gleaner | bark gleaner | 1.000 | insects | 0.000 | bark gleaner | 0.999 | 2.805 | -3.002 | -2.284 | 0.971 | -1.509 | 0.514 |
| *Exilisiciurus exilis* | 3 | bark gleaner | bark gleaner | 1.000 | insects | 0.000 | bark gleaner | 0.998 | 2.892 | -2.868 | -3.339 | -0.498 | -0.695 | 0.058 |
| *Exilisiciurus exilis* | 4 | bark gleaner | bark gleaner | 0.999 | insects | 0.001 | bark gleaner | 0.996 | 3.991 | -3.449 | -1.963 | -1.235 | -0.865 | 1.078 |
| *Exilisiciurus exilis* | 5 | bark gleaner | bark gleaner | 0.992 | insects | 0.008 | bark gleaner | 0.964 | 2.954 | -3.555 | -2.756 | -0.507 | -0.248 | 2.117 |
| *Exilisiciurus exilis* | 6 | bark gleaner | bark gleaner | 1.000 | insects | 0.000 | bark gleaner | 1.000 | 3.584 | -2.601 | -2.846 | -0.573 | 0.257 | -0.715 |
| *Funambulus palmarum* | 1 | fruits | fruits | 0.876 | seeds | 0.103 | fruits | 0.788 | 2.889 | -0.251 | 0.383 | -1.724 | -0.062 | -1.948 |
| *Funambulus palmarum* | 2 | fruits | **insects** | 0.490 | fruits | 0.441 | **insects** | 0.696 | 1.967 | -0.835 | 0.136 | -0.784 | 0.319 | 0.149 |
| *Funambulus palmarum* | 3 | fruits | fruits | 0.504 | insects | 0.325 | **insects** | 0.469 | 2.745 | -1.342 | 0.706 | -0.586 | 0.376 | -0.297 |
| *Funambulus palmarum* | 4 | fruits | fruits | 0.910 | insects | 0.050 | fruits | 0.729 | 2.387 | -0.285 | -0.806 | 0.999 | 0.557 | -0.637 |
| *Funambulus palmarum* | 5 | fruits | **insects** | 0.411 | fruits | 0.342 | **insects** | 0.433 | 3.218 | -1.058 | 0.068 | -0.746 | -0.575 | -0.510 |
| *Funambulus palmarum* | 6 | fruits | fruits | 0.388 | seeds | 0.385 | **seeds** | 0.486 | 2.596 | -1.266 | 0.744 | 0.431 | -0.279 | -0.192 |
| *Funambulus palmarum* | 7 | fruits | fruits | 0.800 | insects | 0.188 | fruits | 0.598 | 2.979 | 0.105 | -0.093 | 0.474 | 0.719 | 0.397 |
| *Funambulus palmarum* | 8 | fruits | fruits | 0.972 | seeds | 0.022 | fruits | 0.956 | 2.425 | 0.890 | 1.116 | -0.193 | 1.415 | -0.403 |
| *Funisciurus congicus* | 1 | nuts | nuts | 0.996 | fruits | 0.003 | nuts | 0.995 | -0.933 | 2.265 | -1.097 | 1.304 | 0.119 | 0.751 |
| *Funisciurus congicus* | 2 | nuts | nuts | 0.965 | fruits | 0.034 | nuts | 0.942 | -0.946 | 1.481 | -0.262 | 1.166 | 0.770 | 0.513 |
| *Funisciurus congicus* | 3 | nuts | nuts | 0.914 | fruits | 0.068 | nuts | 0.864 | -1.499 | 0.226 | 0.458 | 1.235 | 1.036 | 0.089 |
| *Funisciurus congicus* | 4 | nuts | **fruits** | 0.694 | nuts | 0.297 | **fruits** | 0.851 | 0.180 | 0.992 | 0.205 | 1.202 | 2.173 | 0.645 |
| *Funisciurus congicus* | 5 | nuts | nuts | 0.905 | fruits | 0.074 | nuts | 0.818 | -0.864 | -0.090 | -2.290 | 0.173 | 0.204 | -0.215 |
| *Funisciurus congicus* | 6 | nuts | nuts | 0.716 | fruits | 0.276 | nuts | 0.598 | -0.098 | 1.397 | -0.094 | 0.970 | 0.793 | 0.089 |
| *Funisciurus congicus* | 7 | nuts | **fruits** | 0.911 | seeds | 0.061 | **fruits** | 0.928 | 0.636 | 0.642 | 0.579 | 0.134 | 0.932 | -0.677 |
| *Funisciurus congicus* | 8 | nuts | nuts | 0.638 | fruits | 0.351 | **fruits** | 0.560 | -0.221 | 1.045 | 0.172 | 1.302 | 0.964 | -0.889 |
| *Funisciurus congicus* | 9 | nuts | nuts | 0.974 | fruits | 0.026 | nuts | 0.958 | -1.069 | 2.044 | -0.617 | 1.445 | 2.016 | 1.063 |
| *Funisciurus congicus* | 10 | nuts | **fruits** | 0.601 | nuts | 0.343 | **fruits** | 0.728 | -0.416 | -0.161 | -0.452 | 0.484 | 1.479 | 0.320 |
| *Glaucomys volans* | 1 | nuts | nuts | 1.000 | fruits | 0.000 | nuts | 1.000 | -0.505 | 1.611 | -1.897 | 2.260 | -1.556 | 0.885 |
| *Glaucomys volans* | 2 | nuts | nuts | 0.987 | fruits | 0.011 | nuts | 0.983 | -0.591 | 0.989 | -1.349 | 0.768 | -1.084 | 0.015 |
| *Glaucomys volans* | 3 | nuts | nuts | 0.884 | insects | 0.052 | nuts | 0.797 | -0.706 | 1.738 | -1.436 | -1.036 | -1.686 | 0.875 |
| *Glaucomys volans* | 4 | nuts | nuts | 0.990 | fruits | 0.010 | nuts | 0.987 | -0.821 | 1.368 | -1.878 | 0.602 | -0.565 | -0.076 |
| *Glaucomys volans* | 5 | nuts | nuts | 0.997 | leaves | 0.001 | nuts | 0.994 | -1.322 | 0.740 | -2.434 | 0.926 | -1.137 | -0.557 |
| *Glaucomys volans* | 6 | nuts | **fruits** | 0.952 | insects | 0.035 | **fruits** | 0.956 | 0.855 | 0.438 | -1.022 | -0.819 | 0.965 | -0.703 |
| *Glaucomys volans* | 7 | nuts | nuts | 0.919 | fruits | 0.080 | nuts | 0.863 | -1.015 | 2.143 | -1.070 | 0.327 | 0.172 | -1.441 |
| *Heliosciurus gambianus* | 1 | fruits | fruits | 0.995 | nuts | 0.003 | fruits | 0.993 | -0.068 | 0.659 | 0.201 | -0.327 | 2.396 | -2.875 |
| *Heliosciurus gambianus* | 2 | fruits | fruits | 0.914 | nuts | 0.043 | fruits | 0.854 | 1.212 | 1.370 | 0.699 | 0.627 | 0.780 | -0.330 |
| *Heliosciurus gambianus* | 3 | fruits | fruits | 0.992 | seeds | 0.006 | fruits | 0.991 | 2.073 | 0.586 | 0.697 | -0.261 | 1.846 | -1.421 |
| *Heliosciurus gambianus* | 4 | fruits | fruits | 0.994 | nuts | 0.004 | fruits | 0.992 | 0.535 | 1.577 | -0.249 | -0.180 | 3.542 | -0.022 |
| *Heliosciurus gambianus* | 5 | fruits | fruits | 0.982 | seeds | 0.012 | fruits | 0.976 | 0.185 | 0.833 | 0.183 | -0.724 | 1.501 | -1.713 |
| *Heliosciurus gambianus* | 6 | fruits | fruits | 0.866 | nuts | 0.133 | fruits | 0.808 | 0.065 | 2.826 | -0.384 | -0.073 | 1.034 | -1.996 |
| *Heliosciurus gambianus* | 7 | fruits | fruits | 0.953 | nuts | 0.030 | fruits | 0.928 | 1.089 | 1.358 | -0.958 | -0.721 | -0.358 | -1.705 |
| *Heliosciurus gambianus* | 8 | fruits | fruits | 0.718 | nuts | 0.280 | fruits | 0.589 | -0.497 | 1.312 | 0.029 | 0.765 | 2.120 | -0.788 |
| *Heliosciurus gambianus* | 9 | fruits | fruits | 0.982 | nuts | 0.010 | fruits | 0.977 | 0.693 | 1.781 | -0.011 | -0.515 | 2.001 | 0.203 |
| *Heliosciurus gambianus* | 10 | fruits | fruits | 0.996 | seeds | 0.002 | fruits | 0.995 | 0.956 | 1.901 | 0.443 | -1.460 | 2.329 | -0.489 |
| *Hylopetes lepidus* | 1 | unknown | nuts | 0.686 | fruits | 0.313 | - | - | -1.810 | 2.646 | -0.385 | -0.352 | 2.054 | -0.210 |
| *Hylopetes lepidus* | 2 | unknown | nuts | 0.853 | fruits | 0.111 | - | - | -1.966 | 1.108 | -1.320 | -0.386 | 1.387 | 0.169 |
| *Hylopetes lepidus* | 3 | unknown | nuts | 0.919 | fruits | 0.080 | - | - | -1.695 | 2.941 | -0.276 | 0.084 | 1.335 | 0.001 |
| *Hylopetes lepidus* | 4 | unknown | nuts | 0.727 | fruits | 0.269 | - | - | -1.365 | 2.283 | -1.241 | -0.360 | 1.114 | -1.270 |
| *Hylopetes lepidus* | 5 | unknown | fruits | 0.479 | nuts | 0.405 | - | - | -1.003 | 0.562 | -1.022 | -1.174 | -0.462 | -0.701 |
| *Hylopetes lepidus* | 6 | unknown | nuts | 0.904 | leaves | 0.086 | - | - | -2.365 | 1.911 | -0.885 | -0.239 | -0.633 | -1.132 |
| *Hylopetes lepidus* | 7 | unknown | nuts | 0.837 | fruits | 0.145 | - | - | -1.149 | 3.162 | -0.294 | -0.991 | -0.617 | -0.022 |
| *Iomys horsfieldii* | 1 | fruits | **seeds** | 0.588 | fruits | 0.370 | **seeds** | 0.795 | -0.979 | 2.198 | 1.283 | -1.296 | -0.213 | -1.041 |
| *Iomys horsfieldii* | 2 | fruits | **nuts** | 0.822 | fruits | 0.112 | **nuts** | 0.908 | -1.824 | 0.717 | 0.358 | 0.205 | 0.320 | -0.555 |
| *Iomys horsfieldii* | 3 | fruits | **nuts** | 0.510 | seeds | 0.449 | **nuts** | 0.565 | -1.005 | 2.140 | 1.224 | 0.260 | -1.416 | -0.655 |
| *Lariscus insignis* | 1 | fruits | fruits | 0.542 | seeds | 0.438 | **seeds** | 0.573 | 2.454 | 1.039 | 2.103 | -1.426 | 0.478 | 0.156 |
| *Lariscus insignis* | 2 | fruits | fruits | 0.894 | seeds | 0.086 | fruits | 0.851 | 0.967 | 1.934 | 1.149 | -0.985 | 0.871 | 0.613 |
| *Lariscus insignis* | 3 | fruits | fruits | 0.873 | insects | 0.068 | fruits | 0.820 | 1.388 | 0.914 | 0.274 | -0.392 | 0.483 | 0.348 |
| *Lariscus insignis* | 4 | fruits | fruits | 0.923 | seeds | 0.056 | fruits | 0.890 | 1.394 | 1.299 | 1.241 | -0.095 | 1.305 | 0.633 |
| *Lariscus insignis* | 5 | fruits | fruits | 0.811 | seeds | 0.116 | fruits | 0.765 | 1.829 | 0.842 | 1.210 | -0.434 | 0.857 | 0.758 |
| *Lariscus insignis* | 6 | fruits | fruits | 0.751 | insects | 0.161 | fruits | 0.615 | 2.048 | -0.385 | 0.847 | 0.819 | 1.002 | 0.715 |
| *Lariscus insignis* | 7 | fruits | fruits | 0.512 | insects | 0.457 | **insects** | 0.559 | 1.164 | -0.025 | 0.123 | -0.593 | 0.946 | 1.025 |
| *Lariscus insignis* | 8 | fruits | fruits | 0.693 | seeds | 0.299 | fruits | 0.547 | 2.203 | 1.620 | 1.151 | -0.197 | -0.502 | -0.508 |
| *Lariscus insignis* | 9 | fruits | fruits | 0.953 | seeds | 0.026 | fruits | 0.941 | 2.257 | 0.984 | 0.555 | 0.071 | 0.751 | -0.079 |
| *Lariscus insignis* | 10 | fruits | fruits | 0.943 | seeds | 0.053 | fruits | 0.919 | 1.616 | 1.790 | 2.165 | -0.570 | 2.055 | 0.596 |
| *Marmota marmota* | 1 | herbivore ss | herbivore ss | 1.000 | nuts | 0.000 | herbivore ss | 1.000 | -4.403 | -3.539 | 0.699 | 0.619 | 1.564 | 1.136 |
| *Marmota marmota* | 2 | herbivore ss | herbivore ss | 1.000 | seeds | 0.000 | herbivore ss | 1.000 | -3.737 | -3.307 | 1.564 | 0.551 | 0.842 | 0.258 |
| *Marmota marmota* | 3 | herbivore ss | herbivore ss | 1.000 | seeds | 0.000 | herbivore ss | 0.978 | -3.033 | -3.269 | 1.424 | -0.843 | -1.159 | -0.361 |
| *Marmota marmota* | 4 | herbivore ss | herbivore ss | 1.000 | seeds | 0.000 | herbivore ss | 1.000 | -3.861 | -3.585 | 0.862 | 0.360 | 0.915 | -0.712 |
| *Marmota marmota* | 5 | herbivore ss | herbivore ss | 1.000 | seeds | 0.000 | herbivore ss | 1.000 | -4.096 | -3.719 | 1.831 | 0.949 | 1.234 | -0.734 |
| *Marmota marmota* | 6 | herbivore ss | herbivore ss | 1.000 | seeds | 0.000 | herbivore ss | 1.000 | -2.785 | -3.697 | 0.473 | 0.718 | 1.185 | -0.965 |
| *Marmota marmota* | 7 | herbivore ss | herbivore ss | 1.000 | seeds | 0.000 | herbivore ss | 0.998 | -3.635 | -4.305 | 1.912 | 0.480 | 0.546 | -0.536 |
| *Marmota marmota* | 8 | herbivore ss | herbivore ss | 1.000 | leaves | 0.000 | herbivore ss | 1.000 | -4.447 | -2.271 | 1.171 | 0.478 | 1.178 | -1.132 |
| *Marmota marmota* | 9 | herbivore ss | herbivore ss | 1.000 | seeds | 0.000 | herbivore ss | 1.000 | -2.731 | -4.113 | 2.102 | 0.197 | 0.778 | -0.515 |
| *Marmota marmota* | 10 | herbivore ss | herbivore ss | 1.000 | nuts | 0.000 | herbivore ss | 1.000 | -4.131 | -3.104 | 0.839 | 0.580 | 1.257 | 0.918 |
| *Menetes berdmorei* | 1 | seeds | **insects** | 0.897 | fruits | 0.056 | **insects** | 0.950 | 1.292 | -0.772 | 0.681 | -1.186 | 0.357 | 1.701 |
| *Menetes berdmorei* | 2 | seeds | **fruits** | 0.935 | seeds | 0.061 | **fruits** | 0.979 | 1.168 | 1.684 | 1.087 | -2.593 | 0.982 | -0.884 |
| *Menetes berdmorei* | 3 | seeds | **fruits** | 0.436 | seeds | 0.336 | **fruits** | 0.507 | 2.428 | 0.942 | 1.549 | -1.649 | 0.094 | 1.011 |
| *Menetes berdmorei* | 4 | seeds | seeds | 0.488 | fruits | 0.456 | **fruits** | 0.541 | 1.485 | 0.070 | 0.539 | -1.204 | -0.682 | -0.685 |
| *Menetes berdmorei* | 5 | seeds | seeds | 0.891 | fruits | 0.071 | seeds | 0.710 | 1.631 | 0.217 | 2.350 | -0.912 | -0.093 | 1.110 |
| *Menetes berdmorei* | 6 | seeds | seeds | 0.922 | fruits | 0.055 | seeds | 0.873 | 1.413 | 0.285 | 1.920 | -0.044 | -0.747 | 0.934 |
| *Menetes berdmorei* | 7 | seeds | seeds | 0.727 | fruits | 0.265 | seeds | 0.583 | 1.459 | 0.671 | 1.470 | -0.012 | -0.557 | -0.530 |
| *Menetes berdmorei* | 8 | seeds | **fruits** | 0.491 | seeds | 0.469 | **fruits** | 0.673 | 1.853 | -0.021 | 0.423 | -1.901 | -0.876 | -1.386 |
| *Menetes berdmorei* | 9 | seeds | **insects** | 0.667 | bark gleaner | 0.251 | **bark gleaner** | 0.501 | 1.558 | -1.341 | -1.439 | -0.981 | -1.874 | -0.342 |
| *Menetes berdmorei* | 10 | seeds | **insects** | 0.351 | nuts | 0.288 | **insects** | 0.391 | 0.939 | -0.149 | -0.183 | 0.847 | -0.392 | 1.057 |
| *Microsciurus flaviventer* | 1 | insects | insects | 0.858 | bark gleaner | 0.083 | insects | 0.775 | 2.506 | -1.201 | -1.327 | -0.848 | 0.106 | 0.517 |
| *Microsciurus flaviventer* | 2 | insects | insects | 0.688 | bark gleaner | 0.295 | **bark gleaner** | 0.716 | 1.308 | -1.817 | -2.240 | 0.559 | -0.068 | 1.145 |
| *Microsciurus flaviventer* | 3 | insects | insects | 0.838 | fruits | 0.069 | insects | 0.527 | 0.690 | -1.628 | -0.650 | 0.668 | -0.361 | 0.989 |
| *Microsciurus flaviventer* | 4 | insects | insects | 0.917 | fruits | 0.058 | insects | 0.805 | 1.637 | -1.157 | -2.075 | -0.684 | 0.742 | 0.755 |
| *Microsciurus flaviventer* | 5 | insects | insects | 0.630 | fruits | 0.245 | **fruits** | 0.375 | 2.023 | -1.942 | -1.006 | -0.731 | 1.578 | -0.079 |
| *Microsciurus flaviventer* | 6 | insects | insects | 0.955 | fruits | 0.041 | insects | 0.900 | 1.436 | -1.016 | -1.762 | -0.914 | 0.383 | 0.960 |
| *Microsciurus flaviventer* | 7 | insects | insects | 0.950 | nuts | 0.024 | insects | 0.698 | 0.882 | -0.978 | -2.486 | -0.044 | -0.922 | 1.319 |
| *Microsciurus flaviventer* | 8 | insects | **bark gleaner** | 0.592 | insects | 0.397 | **bark gleaner** | 0.847 | 1.739 | -1.270 | -2.678 | -0.524 | -1.140 | 0.307 |
| *Microsciurus flaviventer* | 9 | insects | insects | 0.751 | nuts | 0.207 | **nuts** | 0.587 | 0.904 | -0.991 | -1.561 | 1.197 | -0.248 | 1.601 |
| *Microsciurus flaviventer* | 10 | insects | insects | 0.660 | fruits | 0.292 | **fruits** | 0.559 | 0.881 | -0.288 | -1.612 | -0.061 | 0.779 | 0.879 |
| *Myosciurus pumilio* | 1 | bark gleaner | bark gleaner | 1.000 | insects | 0.000 | bark gleaner | 1.000 | 3.701 | -5.236 | -3.599 | 1.874 | -1.213 | 0.182 |
| *Myosciurus pumilio* | 2 | bark gleaner | bark gleaner | 1.000 | insects | 0.000 | bark gleaner | 1.000 | 3.630 | -4.647 | -4.081 | 1.235 | -2.882 | -0.587 |
| *Nannosciurus melanotis* | 1 | bark gleaner | bark gleaner | 1.000 | insects | 0.000 | bark gleaner | 1.000 | 3.610 | -3.091 | -2.190 | -0.063 | -1.171 | -1.649 |
| *Nannosciurus melanotis* | 2 | bark gleaner | bark gleaner | 1.000 | insects | 0.000 | bark gleaner | 0.999 | 3.423 | -2.478 | -2.494 | -0.266 | -0.100 | -0.398 |
| *Nannosciurus melanotis* | 3 | bark gleaner | bark gleaner | 1.000 | insects | 0.000 | bark gleaner | 1.000 | 3.480 | -3.137 | -1.657 | -0.384 | 0.166 | -1.132 |
| *Nannosciurus melanotis* | 4 | bark gleaner | bark gleaner | 1.000 | insects | 0.000 | bark gleaner | 1.000 | 4.348 | -2.697 | -1.275 | -0.095 | -0.756 | -1.101 |
| *Nannosciurus melanotis* | 5 | bark gleaner | bark gleaner | 1.000 | insects | 0.000 | bark gleaner | 1.000 | 5.586 | -3.251 | -0.756 | 1.477 | -0.612 | -1.621 |
| *Nannosciurus melanotis* | 6 | bark gleaner | bark gleaner | 1.000 | insects | 0.000 | bark gleaner | 1.000 | 3.208 | -3.552 | -3.108 | -0.068 | 1.631 | 0.160 |
| *Nannosciurus melanotis* | 7 | bark gleaner | bark gleaner | 1.000 | insects | 0.000 | bark gleaner | 1.000 | 4.383 | -3.583 | -1.248 | -0.713 | 0.105 | -0.602 |
| *Nannosciurus melanotis* | 8 | bark gleaner | bark gleaner | 0.999 | insects | 0.000 | bark gleaner | 0.998 | 4.214 | -2.888 | -1.305 | 0.238 | 0.696 | 0.173 |
| *Nannosciurus melanotis* | 9 | bark gleaner | bark gleaner | 1.000 | insects | 0.000 | bark gleaner | 1.000 | 4.208 | -2.758 | -2.509 | 0.138 | -1.259 | -1.831 |
| *Nannosciurus melanotis* | 10 | bark gleaner | bark gleaner | 1.000 | insects | 0.000 | bark gleaner | 1.000 | 3.150 | -3.257 | -2.156 | 0.654 | -0.137 | -0.236 |
| *Paraxerus ochraceus* | 1 | fruits | **nuts** | 0.539 | fruits | 0.444 | **nuts** | 0.688 | -0.659 | 1.349 | -0.691 | 0.022 | 1.554 | 0.780 |
| *Paraxerus ochraceus* | 2 | fruits | fruits | 0.763 | nuts | 0.222 | fruits | 0.637 | -0.718 | 1.647 | -0.432 | -0.560 | 1.715 | 0.531 |
| *Paraxerus ochraceus* | 3 | fruits | fruits | 0.955 | nuts | 0.039 | fruits | 0.926 | -0.568 | 3.075 | 0.755 | -0.454 | 1.805 | -1.875 |
| *Paraxerus ochraceus* | 4 | fruits | fruits | 0.975 | seeds | 0.019 | fruits | 0.970 | 0.724 | 2.363 | 0.814 | -0.885 | 1.162 | -0.628 |
| *Paraxerus ochraceus* | 5 | fruits | fruits | 0.435 | seeds | 0.342 | **seeds** | 0.476 | -0.386 | 2.871 | 0.340 | -1.503 | -1.174 | -0.007 |
| *Paraxerus ochraceus* | 6 | fruits | fruits | 0.847 | nuts | 0.082 | fruits | 0.761 | 0.532 | 1.838 | -0.411 | -1.008 | -0.520 | -0.530 |
| *Paraxerus ochraceus* | 7 | fruits | fruits | 0.899 | nuts | 0.072 | fruits | 0.859 | 0.197 | 2.804 | 0.491 | -0.695 | 0.589 | -0.220 |
| *Paraxerus ochraceus* | 8 | fruits | fruits | 0.841 | seeds | 0.153 | fruits | 0.768 | 1.385 | 1.108 | 0.730 | -0.619 | 0.021 | -1.165 |
| *Paraxerus ochraceus* | 9 | fruits | fruits | 0.896 | seeds | 0.058 | fruits | 0.838 | 0.383 | 0.736 | 0.695 | 0.281 | 1.135 | -0.469 |
| *Paraxerus ochraceus* | 10 | fruits | fruits | 0.588 | seeds | 0.315 | **seeds** | 0.438 | -0.111 | 0.815 | 0.944 | -0.133 | 0.432 | 0.166 |
| *Petaurillus kinlochii* | - | unknown | nuts | 0.624 | fruits | 0.374 | - | - | 0.623 | 0.839 | -1.683 | 1.441 | 1.500 | -0.528 |
| *Petaurista petaurista* | 1 | leaves | leaves | 1.000 | nuts | 0.000 | leaves | 1.000 | -3.702 | 2.228 | -2.535 | -2.173 | 0.179 | -0.278 |
| *Petaurista petaurista* | 2 | leaves | leaves | 1.000 | nuts | 0.000 | leaves | 1.000 | -3.986 | -0.274 | -3.362 | -0.969 | 0.433 | 0.284 |
| *Petaurista petaurista* | 3 | leaves | leaves | 0.997 | nuts | 0.003 | leaves | 0.979 | -2.912 | 0.414 | -2.778 | -1.139 | 0.771 | 0.656 |
| *Petaurista petaurista* | 4 | leaves | leaves | 1.000 | nuts | 0.000 | leaves | 0.999 | -2.984 | 1.508 | -2.848 | -1.573 | -0.734 | -0.123 |
| *Petaurista petaurista* | 5 | leaves | leaves | 0.999 | nuts | 0.001 | leaves | 0.998 | -3.120 | 1.790 | -3.058 | -0.976 | -0.575 | -1.107 |
| *Petaurista petaurista* | 6 | leaves | leaves | 1.000 | nuts | 0.000 | leaves | 1.000 | -3.815 | 1.829 | -3.567 | -1.661 | -0.375 | 0.443 |
| *Petaurista petaurista* | 7 | leaves | leaves | 1.000 | nuts | 0.000 | leaves | 1.000 | -3.703 | 1.062 | -3.805 | -2.319 | 0.624 | -1.878 |
| *Petaurista petaurista* | 8 | leaves | leaves | 1.000 | nuts | 0.000 | leaves | 1.000 | -4.420 | 1.224 | -2.614 | -0.912 | 0.218 | -0.770 |
| *Petaurista petaurista* | 9 | leaves | leaves | 0.827 | nuts | 0.165 | **nuts** | 0.789 | -2.421 | 0.466 | -3.005 | -0.525 | 0.716 | 2.140 |
| *Petaurista petaurista* | 10 | leaves | leaves | 1.000 | nuts | 0.000 | leaves | 1.000 | -4.212 | 1.464 | -3.095 | -1.878 | 0.799 | -0.895 |
| *Petinomys genibarbis* | 1 | unknown | nuts | 0.686 | seeds | 0.198 | - | - | -0.793 | 1.049 | -0.458 | -0.558 | -1.689 | -0.655 |
| *Petinomys genibarbis* | 2 | unknown | fruits | 0.734 | nuts | 0.255 | - | - | -0.724 | 2.157 | 0.210 | -0.309 | 1.470 | 0.061 |
| *Petinomys genibarbis* | 3 | unknown | nuts | 0.987 | fruits | 0.007 | - | - | -2.236 | 1.641 | -1.328 | 0.469 | 0.636 | -0.409 |
| *Petinomys genibarbis* | 4 | unknown | nuts | 0.999 | fruits | 0.001 | - | - | -2.512 | 2.890 | -0.475 | 0.802 | -0.093 | 0.244 |
| *Petinomys genibarbis* | 5 | unknown | fruits | 0.740 | nuts | 0.220 | - | - | -0.573 | 2.141 | -0.027 | -0.806 | 0.241 | -0.858 |
| *Petinomys genibarbis* | 6 | unknown | leaves | 1.000 | herbivore ss | 0.000 | - | - | -3.222 | -0.838 | -2.734 | -2.619 | -0.205 | -1.482 |
| *Petinomys genibarbis* | 7 | unknown | fruits | 0.548 | nuts | 0.442 | - | - | -1.047 | 1.006 | 0.076 | 0.405 | 1.568 | -0.800 |
| *Petinomys genibarbis* | 8 | unknown | fruits | 0.514 | nuts | 0.462 | - | - | -0.550 | 1.616 | -0.011 | 0.172 | 0.477 | -1.144 |
| *Petinomys genibarbis* | 9 | unknown | nuts | 0.950 | fruits | 0.046 | - | - | -1.763 | 2.295 | -1.182 | -0.028 | 0.796 | -0.684 |
| *Petinomys genibarbis* | 10 | unknown | nuts | 0.833 | fruits | 0.163 | - | - | -1.445 | 1.941 | -0.167 | 0.052 | 1.100 | 0.109 |
| *Prosciurillus leucomus* | 1 | fruits | fruits | 0.780 | insects | 0.159 | fruits | 0.627 | 2.240 | -0.143 | -0.986 | 0.182 | -0.474 | -0.625 |
| *Prosciurillus leucomus* | 2 | fruits | fruits | 0.962 | insects | 0.036 | fruits | 0.927 | 2.022 | 0.037 | -0.500 | -1.029 | 1.973 | -0.634 |
| *Prosciurillus leucomus* | 3 | fruits | fruits | 0.930 | insects | 0.069 | fruits | 0.867 | 2.591 | 0.490 | 0.075 | -0.544 | 2.143 | 0.381 |
| *Prosciurillus leucomus* | 4 | fruits | fruits | 0.841 | insects | 0.148 | fruits | 0.562 | 3.075 | -1.116 | 0.177 | -0.777 | 1.976 | -0.472 |
| *Prosciurillus leucomus* | 5 | fruits | fruits | 0.831 | seeds | 0.134 | fruits | 0.721 | 3.219 | 0.562 | 0.308 | -0.464 | -0.773 | -1.001 |
| *Prosciurillus leucomus* | 6 | fruits | fruits | 0.975 | insects | 0.024 | fruits | 0.965 | 1.518 | 0.855 | -0.788 | -0.684 | 2.684 | 0.093 |
| *Prosciurillus leucomus* | 7 | fruits | fruits | 0.988 | seeds | 0.007 | fruits | 0.985 | 1.310 | -0.084 | 0.349 | -0.094 | 2.070 | -1.039 |
| *Prosciurillus leucomus* | 8 | fruits | fruits | 0.692 | insects | 0.284 | fruits | 0.548 | 2.639 | -0.034 | -0.854 | -0.134 | -0.111 | -0.181 |
| *Protoxerus stangeri* | 1 | nuts | nuts | 0.998 | seeds | 0.001 | nuts | 0.997 | -0.254 | 2.231 | 0.837 | 3.128 | -1.895 | 0.743 |
| *Protoxerus stangeri* | 2 | nuts | nuts | 0.999 | fruits | 0.001 | nuts | 0.999 | -0.469 | 2.930 | -1.374 | 1.936 | -0.485 | -0.065 |
| *Protoxerus stangeri* | 3 | nuts | nuts | 0.999 | fruits | 0.001 | nuts | 0.998 | -0.399 | 1.647 | -0.330 | 2.311 | -1.056 | 0.756 |
| *Protoxerus stangeri* | 4 | nuts | nuts | 1.000 | fruits | 0.000 | nuts | 1.000 | 0.059 | 2.360 | -0.436 | 4.339 | 0.229 | 1.243 |
| *Protoxerus stangeri* | 5 | nuts | nuts | 1.000 | seeds | 0.000 | nuts | 1.000 | -0.694 | 1.308 | 0.136 | 3.396 | -1.626 | 0.830 |
| *Protoxerus stangeri* | 6 | nuts | nuts | 1.000 | fruits | 0.000 | nuts | 1.000 | -0.770 | 3.464 | 0.522 | 3.883 | -0.004 | 0.251 |
| *Protoxerus stangeri* | 7 | nuts | nuts | 0.999 | fruits | 0.001 | nuts | 0.999 | -0.620 | 2.534 | -0.569 | 2.350 | -0.653 | 0.244 |
| *Protoxerus stangeri* | 8 | nuts | nuts | 1.000 | fruits | 0.000 | nuts | 1.000 | -0.736 | 1.466 | -0.165 | 4.096 | -0.251 | 1.059 |
| *Protoxerus stangeri* | 9 | nuts | nuts | 1.000 | fruits | 0.000 | nuts | 1.000 | -1.027 | 2.804 | -1.417 | 2.894 | -1.795 | 0.687 |
| *Protoxerus stangeri* | 10 | nuts | nuts | 1.000 | fruits | 0.000 | nuts | 1.000 | 0.094 | 2.364 | -0.451 | 4.182 | 0.173 | 1.016 |
| *Pteromys volans* | - | leaves | leaves | 0.984 | nuts | 0.016 | leaves | 0.518 | -2.744 | 0.317 | -3.225 | -0.028 | -0.468 | -1.724 |
| *Pteromyscus pulverulentus* | - | unknown | fruits | 0.426 | nuts | 0.335 | - | - | -1.302 | 1.839 | -0.496 | -1.847 | 0.169 | 1.311 |
| *Ratufa bicolor* | 1 | nuts | nuts | 0.960 | fruits | 0.040 | nuts | 0.941 | -1.268 | 2.267 | -1.516 | 0.635 | 1.207 | -0.777 |
| *Ratufa bicolor* | 2 | nuts | nuts | 0.998 | fruits | 0.002 | nuts | 0.997 | -1.227 | 0.857 | -1.448 | 1.688 | -0.492 | -0.901 |
| *Ratufa bicolor* | 3 | nuts | nuts | 0.977 | fruits | 0.023 | nuts | 0.965 | -1.034 | 2.187 | -0.558 | 1.300 | 0.889 | -0.344 |
| *Ratufa bicolor* | 4 | nuts | nuts | 0.972 | fruits | 0.027 | nuts | 0.926 | -1.228 | 1.775 | -0.759 | 0.372 | -0.014 | 0.102 |
| *Ratufa bicolor* | 5 | nuts | nuts | 0.973 | fruits | 0.026 | nuts | 0.949 | -0.925 | 2.008 | -1.092 | 0.358 | -0.657 | -0.803 |
| *Ratufa bicolor* | 6 | nuts | nuts | 0.991 | fruits | 0.009 | nuts | 0.984 | -1.300 | -0.284 | -1.521 | 1.765 | 0.309 | -1.397 |
| *Ratufa bicolor* | 7 | nuts | nuts | 0.800 | leaves | 0.198 | **leaves** | 0.643 | -2.386 | 1.615 | -1.924 | 0.003 | -0.240 | -0.447 |
| *Ratufa bicolor* | 8 | nuts | nuts | 0.991 | fruits | 0.009 | nuts | 0.986 | -0.860 | 2.494 | -0.949 | 1.507 | 0.046 | -1.397 |
| *Ratufa bicolor* | 9 | nuts | nuts | 0.960 | fruits | 0.039 | nuts | 0.920 | -0.993 | 1.520 | -0.683 | 1.262 | 0.320 | -1.706 |
| *Ratufa bicolor* | 10 | nuts | nuts | 0.915 | fruits | 0.076 | nuts | 0.865 | -0.616 | 1.607 | -0.289 | 0.743 | -0.331 | -0.833 |
| *Rheithrosciurus macrotis* | 1 | fruits | **nuts** | 0.988 | fruits | 0.012 | **nuts** | 1.000 | 0.866 | 3.172 | -0.887 | 3.238 | 0.271 | -2.343 |
| *Rheithrosciurus macrotis* | 2 | fruits | fruits | 0.963 | nuts | 0.027 | fruits | 0.862 | 2.048 | 1.606 | 0.137 | 1.084 | 1.739 | 0.624 |
| *Rhinosciurus laticaudatus* | 1 | insects | insects | 1.000 | fruits | 0.000 | insects | 0.999 | 0.944 | -1.743 | -0.214 | -1.648 | 1.000 | 3.658 |
| *Rhinosciurus laticaudatus* | 2 | insects | insects | 0.999 | fruits | 0.000 | insects | 0.999 | 1.574 | -0.147 | -0.138 | -2.704 | 0.887 | 3.926 |
| *Rhinosciurus laticaudatus* | 3 | insects | insects | 1.000 | fruits | 0.000 | insects | 1.000 | 1.605 | -0.596 | 0.123 | -3.995 | 0.965 | 3.843 |
| *Rhinosciurus laticaudatus* | 4 | insects | insects | 0.989 | seeds | 0.010 | insects | 0.961 | 1.113 | 0.261 | 0.994 | -3.038 | -1.129 | 3.686 |
| *Rhinosciurus laticaudatus* | 5 | insects | insects | 1.000 | fruits | 0.000 | insects | 1.000 | 1.222 | -1.754 | -0.831 | -3.153 | 0.230 | 3.660 |
| *Rhinosciurus laticaudatus* | 6 | insects | insects | 0.994 | seeds | 0.006 | insects | 0.970 | 1.270 | -0.231 | 0.366 | -1.665 | -1.323 | 3.454 |
| *Rhinosciurus laticaudatus* | 7 | insects | insects | 0.999 | seeds | 0.001 | insects | 0.998 | 1.196 | -0.487 | 0.456 | -2.690 | -0.926 | 4.145 |
| *Sciurillus pusillus* | 1 | bark gleaner | bark gleaner | 1.000 | insects | 0.000 | bark gleaner | 1.000 | 4.948 | -1.744 | -2.090 | 1.854 | -1.984 | -1.404 |
| *Sciurillus pusillus* | 2 | bark gleaner | bark gleaner | 1.000 | insects | 0.000 | bark gleaner | 1.000 | 4.109 | -2.796 | -3.134 | -1.138 | -0.821 | -3.146 |
| *Sciurillus pusillus* | 3 | bark gleaner | bark gleaner | 1.000 | insects | 0.000 | bark gleaner | 1.000 | 3.329 | -2.400 | -1.688 | 1.581 | -0.084 | -1.009 |
| *Sciurillus pusillus* | 4 | bark gleaner | bark gleaner | 1.000 | insects | 0.000 | bark gleaner | 1.000 | 2.880 | -2.453 | -3.055 | 1.169 | 1.310 | -0.960 |
| *Sciurillus pusillus* | 5 | bark gleaner | bark gleaner | 1.000 | fruits | 0.000 | bark gleaner | 1.000 | 4.056 | -2.392 | -1.322 | 2.004 | -0.421 | -1.364 |
| *Sciurillus pusillus* | 6 | bark gleaner | bark gleaner | 1.000 | insects | 0.000 | bark gleaner | 0.999 | 3.195 | -2.322 | -2.711 | -0.173 | -0.492 | -0.354 |
| *Sciurillus pusillus* | 7 | bark gleaner | bark gleaner | 0.995 | fruits | 0.003 | bark gleaner | 0.949 | 3.189 | -1.431 | -1.622 | 1.943 | 0.109 | -0.355 |
| *Sciurotamias davidianus* | 1 | seeds | seeds | 0.792 | nuts | 0.189 | seeds | 0.573 | -1.229 | 1.079 | 1.835 | 0.513 | -0.869 | 0.367 |
| *Sciurotamias davidianus* | 2 | seeds | seeds | 1.000 | insects | 0.000 | seeds | 1.000 | 0.426 | -0.986 | 3.672 | -0.885 | -2.435 | 0.622 |
| *Sciurotamias davidianus* | 3 | seeds | seeds | 0.998 | fruits | 0.001 | seeds | 0.997 | 0.839 | 0.257 | 3.433 | -1.007 | -0.982 | 1.097 |
| *Sciurotamias davidianus* | 4 | seeds | seeds | 0.993 | fruits | 0.006 | seeds | 0.990 | 0.225 | 0.378 | 2.602 | 0.139 | -1.079 | 0.365 |
| *Sciurotamias davidianus* | 5 | seeds | seeds | 0.987 | fruits | 0.009 | seeds | 0.982 | -0.404 | 1.032 | 2.659 | -0.041 | -0.753 | 0.765 |
| *Sciurotamias davidianus* | 6 | seeds | seeds | 0.999 | fruits | 0.001 | seeds | 0.998 | 1.206 | -0.135 | 2.112 | -1.678 | -2.651 | -1.101 |
| *Sciurus vulgaris* | 1 | nuts | **seeds** | 0.436 | fruits | 0.350 | **seeds** | 0.571 | 0.196 | 1.165 | 1.164 | 0.707 | -0.223 | -0.452 |
| *Sciurus vulgaris* | 2 | nuts | nuts | 0.975 | fruits | 0.015 | nuts | 0.963 | -0.911 | 2.023 | 0.531 | 1.376 | -0.523 | -0.484 |
| *Sciurus vulgaris* | 3 | nuts | nuts | 0.990 | fruits | 0.010 | nuts | 0.985 | -1.035 | 1.859 | -1.073 | 0.662 | -0.422 | 0.133 |
| *Sciurus vulgaris* | 4 | nuts | nuts | 0.971 | fruits | 0.024 | nuts | 0.958 | -1.173 | 1.099 | 0.522 | 1.643 | 0.866 | 0.559 |
| *Sciurus vulgaris* | 5 | nuts | nuts | 0.976 | seeds | 0.010 | nuts | 0.947 | -0.503 | -0.018 | -0.907 | 1.060 | -1.647 | 0.525 |
| *Sciurus vulgaris* | 6 | nuts | nuts | 0.973 | fruits | 0.027 | nuts | 0.961 | -1.432 | 1.071 | -1.114 | 1.206 | 1.571 | -0.075 |
| *Sciurus vulgaris* | 7 | nuts | nuts | 1.000 | seeds | 0.000 | nuts | 1.000 | -1.751 | 1.243 | 0.059 | 3.047 | -1.782 | 0.550 |
| *Sciurus vulgaris* | 8 | nuts | nuts | 0.976 | fruits | 0.024 | nuts | 0.961 | -1.151 | 1.844 | -0.874 | 0.682 | 0.660 | 0.616 |
| *Sciurus vulgaris* | 9 | nuts | nuts | 0.999 | fruits | 0.001 | nuts | 0.998 | -1.269 | 2.117 | 0.142 | 2.051 | -0.356 | 0.511 |
| *Sciurus vulgaris* | 10 | nuts | nuts | 0.993 | fruits | 0.007 | nuts | 0.990 | -1.353 | 1.180 | -1.241 | 0.926 | 0.009 | 0.083 |
| *Spermophilopsis leptodactylus* | - | herbivore ss | herbivore ss | 1.000 | seeds | 0.000 | herbivore ss | 0.997 | -0.785 | -4.324 | 0.325 | 1.354 | 1.178 | -1.331 |
| *Sundasciurus altitudinis* | 1 | seeds | **fruits** | 0.517 | seeds | 0.445 | **fruits** | 0.733 | 1.495 | 2.540 | 1.703 | 0.074 | -0.294 | 1.009 |
| *Sundasciurus altitudinis* | 2 | seeds | **fruits** | 0.871 | insects | 0.062 | **fruits** | 0.883 | 0.808 | 0.485 | 0.306 | 0.129 | 1.116 | 0.540 |
| *Sundasciurus altitudinis* | 3 | seeds | **fruits** | 0.848 | insects | 0.066 | **fruits** | 0.862 | 1.527 | 1.692 | 0.151 | -0.118 | 0.085 | 0.760 |
| *Sundasciurus altitudinis* | 4 | seeds | seeds | 0.912 | fruits | 0.079 | seeds | 0.847 | 1.575 | 1.043 | 2.198 | 0.870 | -0.752 | 0.477 |
| *Sundasciurus altitudinis* | 5 | seeds | **fruits** | 0.858 | nuts | 0.078 | **fruits** | 0.880 | 0.893 | 1.478 | 0.411 | 0.178 | 0.373 | -0.067 |
| *Sundasciurus altitudinis* | 6 | seeds | **fruits** | 0.728 | seeds | 0.202 | **fruits** | 0.823 | 0.369 | 1.473 | 0.550 | -0.588 | -0.074 | -0.087 |
| *Sundasciurus altitudinis* | 7 | seeds | **fruits** | 0.804 | seeds | 0.185 | **fruits** | 0.862 | 1.650 | 0.832 | 1.225 | -0.216 | 0.511 | -0.340 |
| *Sundasciurus altitudinis* | 8 | seeds | **fruits** | 0.777 | seeds | 0.197 | **fruits** | 0.833 | 1.569 | 1.622 | 1.157 | -0.556 | 0.179 | 0.395 |
| *Sundasciurus altitudinis* | 9 | seeds | seeds | 0.924 | fruits | 0.068 | seeds | 0.836 | 1.614 | 1.595 | 1.738 | 0.007 | -1.542 | 0.478 |
| *Sundasciurus altitudinis* | 10 | seeds | **fruits** | 0.856 | seeds | 0.072 | **fruits** | 0.892 | 1.776 | 2.251 | 1.053 | 0.795 | 0.273 | 0.467 |
| *Tamias striatus* | 1 | seeds | seeds | 0.988 | fruits | 0.010 | seeds | 0.980 | -0.533 | 0.661 | 2.417 | 0.083 | -1.109 | -2.050 |
| *Tamias striatus* | 2 | seeds | seeds | 0.960 | nuts | 0.039 | seeds | 0.863 | -1.474 | 0.548 | 1.746 | 0.303 | -2.348 | -0.522 |
| *Tamias striatus* | 3 | seeds | seeds | 0.994 | fruits | 0.006 | seeds | 0.992 | 0.974 | -0.298 | 2.999 | 0.425 | -0.746 | -0.311 |
| *Tamias striatus* | 4 | seeds | seeds | 0.918 | fruits | 0.034 | seeds | 0.729 | -0.680 | -1.151 | 2.601 | 0.903 | 1.076 | 1.278 |
| *Tamias striatus* | 5 | seeds | seeds | 0.992 | fruits | 0.007 | seeds | 0.988 | 2.376 | 1.023 | 2.432 | -0.836 | -2.184 | 0.032 |
| *Tamias striatus* | 6 | seeds | seeds | 0.994 | fruits | 0.006 | seeds | 0.991 | 0.227 | -1.080 | 2.446 | 0.273 | -0.718 | -0.739 |
| *Tamias striatus* | 7 | seeds | seeds | 1.000 | fruits | 0.000 | seeds | 1.000 | 0.075 | -1.123 | 3.956 | -0.011 | -2.185 | 0.027 |
| *Tamias striatus* | 8 | seeds | seeds | 0.998 | fruits | 0.002 | seeds | 0.998 | 0.496 | 1.111 | 3.057 | -0.682 | -1.747 | -0.357 |
| *Tamias striatus* | 9 | seeds | seeds | 1.000 | fruits | 0.000 | **bark gleaner** | 1.000 | 0.669 | 0.288 | 3.053 | -0.923 | -2.723 | -0.733 |
| *Tamias striatus* | 10 | seeds | seeds | 0.999 | insects | 0.001 | seeds | 0.998 | 0.300 | -1.371 | 3.249 | -0.914 | -1.951 | 1.290 |
| *Tamiasciurus hudsonicus* | 1 | nuts | nuts | 0.999 | fruits | 0.001 | nuts | 0.999 | -0.293 | 1.019 | -1.437 | 2.694 | -0.470 | 0.144 |
| *Tamiasciurus hudsonicus* | 2 | nuts | nuts | 0.999 | fruits | 0.001 | nuts | 0.999 | 0.237 | 1.455 | -1.578 | 2.225 | -1.519 | 1.360 |
| *Tamiasciurus hudsonicus* | 3 | nuts | nuts | 0.995 | fruits | 0.004 | nuts | 0.992 | 0.381 | 2.349 | -0.515 | 2.234 | 0.129 | 2.219 |
| *Tamiasciurus hudsonicus* | 4 | nuts | nuts | 0.975 | fruits | 0.023 | nuts | 0.966 | 0.023 | 0.098 | -0.703 | 2.310 | 0.544 | 0.544 |
| *Tamiasciurus hudsonicus* | 5 | nuts | nuts | 0.994 | fruits | 0.006 | nuts | 0.991 | 0.262 | 2.358 | -1.394 | 1.875 | 0.241 | 1.282 |
| *Tamiasciurus hudsonicus* | 6 | nuts | nuts | 0.998 | fruits | 0.001 | nuts | 0.997 | -0.232 | 1.355 | -1.334 | 1.512 | -1.565 | 1.273 |
| *Tamiasciurus hudsonicus* | 7 | nuts | nuts | 0.992 | fruits | 0.007 | nuts | 0.990 | 0.648 | 1.617 | -0.065 | 2.903 | -0.679 | -0.121 |
| *Tamiasciurus hudsonicus* | 8 | nuts | nuts | 0.998 | fruits | 0.002 | nuts | 0.998 | -0.783 | 1.240 | -0.890 | 2.591 | 0.886 | 1.081 |
| *Tamiasciurus hudsonicus* | 9 | nuts | nuts | 0.907 | seeds | 0.090 | nuts | 0.740 | -0.755 | 0.989 | 1.133 | 1.736 | -1.534 | 1.344 |
| *Tamiasciurus hudsonicus* | 10 | nuts | nuts | 0.999 | fruits | 0.001 | nuts | 0.998 | -0.610 | 1.139 | -0.597 | 2.182 | -0.550 | 1.654 |
| *Tamiops mcclellandii* | 1 | insects | **fruits** | 0.574 | insects | 0.359 | **fruits** | 0.772 | 0.401 | 1.466 | 0.759 | -1.736 | 0.992 | 1.804 |
| *Tamiops mcclellandii* | 2 | insects | insects | 0.466 | fruits | 0.296 | **fruits** | 0.421 | 2.764 | 0.751 | -0.273 | -1.073 | -2.040 | 0.123 |
| *Tamiops mcclellandii* | 3 | insects | **seeds** | 0.476 | fruits | 0.420 | **seeds** | 0.562 | 1.944 | -0.006 | 1.644 | -1.636 | 0.467 | 0.235 |
| *Tamiops mcclellandii* | 4 | insects | **fruits** | 0.960 | seeds | 0.038 | **fruits** | 0.964 | 2.282 | -0.346 | 0.887 | -0.497 | 1.270 | -2.040 |
| *Tamiops mcclellandii* | 5 | insects | **fruits** | 0.908 | insects | 0.087 | **fruits** | 0.958 | 1.433 | 0.889 | -0.690 | -1.327 | 0.967 | -0.062 |
| *Trogopterus xantiphes* | 1 | leaves | leaves | 1.000 | herbivore ss | 0.000 | leaves | 1.000 | -4.795 | 0.662 | -1.555 | -3.259 | -1.204 | -1.413 |
| *Trogopterus xantiphes* | 2 | leaves | leaves | 1.000 | herbivore ss | 0.000 | leaves | 1.000 | -5.212 | 1.085 | -0.495 | -3.122 | -1.065 | -0.218 |
| *Trogopterus xantiphes* | 3 | leaves | leaves | 1.000 | nuts | 0.000 | leaves | 1.000 | -5.192 | 1.688 | -1.257 | -3.425 | -1.588 | -0.858 |
| *Trogopterus xantiphes* | 4 | leaves | leaves | 1.000 | herbivore ss | 0.000 | leaves | 1.000 | -5.193 | 0.196 | -2.028 | -3.138 | -1.846 | -0.288 |
| *Trogopterus xantiphes* | 5 | leaves | leaves | 1.000 | nuts | 0.000 | **nuts** | 0.333 | -5.084 | 1.323 | -3.900 | -3.847 | -1.637 | -1.585 |
| *Trogopterus xantiphes* | 6 | leaves | leaves | 1.000 | nuts | 0.000 | leaves | 1.000 | -5.279 | 1.202 | -2.730 | -2.516 | -0.387 | -0.527 |
| *Urocitellus undulatus* | 1 | herbivore ss | **nuts** | 0.784 | fruits | 0.168 | **nuts** | 0.767 | 0.066 | 1.072 | -0.692 | 0.290 | -0.522 | 0.583 |
| *Urocitellus undulatus* | 2 | herbivore ss | herbivore ss | 1.000 | leaves | 0.000 | herbivore ss | 1.000 | -5.139 | -2.778 | 0.016 | 0.250 | 1.141 | 0.881 |
| *Urocitellus undulatus* | 3 | herbivore ss | herbivore ss | 1.000 | nuts | 0.000 | herbivore ss | 1.000 | -4.557 | -3.766 | 0.403 | 1.507 | -0.304 | -0.042 |
| *Urocitellus undulatus* | 4 | herbivore ss | herbivore ss | 1.000 | nuts | 0.000 | herbivore ss | 1.000 | -4.550 | -4.165 | 0.806 | 2.267 | -0.136 | -0.190 |
| *Urocitellus undulatus* | 5 | herbivore ss | herbivore ss | 1.000 | seeds | 0.000 | herbivore ss | 1.000 | -1.565 | -5.196 | 1.509 | -0.550 | 0.972 | -1.060 |
| *Urocitellus undulatus* | 6 | herbivore ss | herbivore ss | 1.000 | nuts | 0.000 | herbivore ss | 1.000 | -4.166 | -2.936 | 1.251 | 0.142 | 2.029 | 2.241 |
| *Urocitellus undulatus* | 7 | herbivore ss | herbivore ss | 1.000 | seeds | 0.000 | herbivore ss | 1.000 | -3.513 | -3.800 | 1.015 | 0.167 | 0.318 | -0.627 |
| *Urocitellus undulatus* | 8 | herbivore ss | herbivore ss | 0.999 | seeds | 0.001 | herbivore ss | 0.972 | -3.076 | -1.329 | 3.102 | 0.323 | 2.243 | -2.026 |
| *Urocitellus undulatus* | 9 | herbivore ss | herbivore ss | 1.000 | seeds | 0.000 | herbivore ss | 1.000 | -3.962 | -3.523 | 1.257 | 0.645 | -0.282 | 0.050 |
| *Urocitellus undulatus* | 10 | herbivore ss | herbivore ss | 1.000 | seeds | 0.000 | **insects** | 1.000 | -4.288 | -4.365 | 1.505 | 1.126 | 1.806 | 0.241 |
| *Xerus erythropus* | 1 | seeds | seeds | 0.999 | fruits | 0.001 | seeds | 0.999 | -0.379 | -0.661 | 3.130 | -1.629 | -1.225 | -0.706 |
| *Xerus erythropus* | 2 | seeds | seeds | 0.999 | fruits | 0.001 | seeds | 0.999 | -0.260 | -0.733 | 3.146 | -2.239 | -1.159 | -1.041 |
| *Xerus erythropus* | 3 | seeds | seeds | 0.999 | fruits | 0.001 | seeds | 0.999 | 0.132 | -1.137 | 2.627 | -1.626 | -1.494 | -1.021 |
| *Xerus erythropus* | 4 | seeds | seeds | 1.000 | fruits | 0.000 | seeds | 1.000 | -0.371 | -1.117 | 3.711 | -0.757 | -1.113 | -0.558 |
| *Xerus erythropus* | 5 | seeds | seeds | 0.995 | fruits | 0.005 | seeds | 0.992 | -0.883 | -0.514 | 3.327 | -0.987 | 0.418 | -0.178 |
| *Xerus erythropus* | 6 | seeds | seeds | 0.999 | fruits | 0.001 | seeds | 0.999 | 0.624 | 0.094 | 4.308 | -0.287 | -0.690 | -0.778 |
| *Xerus erythropus* | 7 | seeds | seeds | 0.998 | fruits | 0.002 | seeds | 0.998 | 0.552 | -1.001 | 3.308 | -0.235 | -0.652 | -0.303 |
| *Xerus erythropus* | 8 | seeds | seeds | 0.998 | fruits | 0.002 | seeds | 0.998 | -0.266 | -0.263 | 3.397 | -0.754 | -0.468 | 0.030 |
| *Xerus erythropus* | 9 | seeds | seeds | 0.997 | fruits | 0.003 | seeds | 0.997 | -0.402 | -0.747 | 2.686 | -1.401 | -0.767 | -0.702 |
| *Xerus erythropus* | 10 | seeds | seeds | 1.000 | fruits | 0.000 | seeds | 1.000 | -0.771 | -0.291 | 4.029 | -1.074 | -0.879 | -0.532 |
| *Douglassciurus jeffersoni* | - | unknown | nuts | 0.904 | fruits | 0.068 | - | - | -1.371 | 2.690 | 2.067 | 1.851 | 1.086 | -1.143 |
