# Supplementary material for: Conservatism and Adaptability during Squirrel Radiation: What Is Mandible Shape Telling Us?
Source: PLoS One. 2013 Apr 4;8(4):e61298. doi: 10.1371/journal.pone.0061298 (PMC3617180; doi:10.1371/journal.pone.0061298)
Supplement: Table S4 — Summary of classification results of Canonical Variates Analysis (CVA) of squirrel mandible shape using the dietary categories of Michaux et al . [16] as grouping variable. (DOCX) [file pone.0061298.s009.docx]

**Table S4. Summary of classification results of Canonical Variates Analysis (CVA) of squirrel mandible shape using the dietary categories of Michaux *et al.* [16] as grouping variable.**

|  | Omnivore animal-dominated | Omnivore plant-dominated | Plant-dominated |
| --- | --- | --- | --- |
| Omnivore animal-dominated | 71.4  (71.4) | 28.6  (28.6) | 0.0  (0.0) |
| Omnivore plant-dominated | 1.0  (3.1) | 92.7  (89.1) | 6.2  (7.8) |
| Plant-dominated | 2.7  (4.1) | 37.0  (41.1) | 60.3  (54.8) |

The numbers refer to the percent of cases assigned to each category. The results after cross-validation are in brackets.
