# Supplementary material for: Conservatism and Adaptability during Squirrel Radiation: What Is Mandible Shape Telling Us?
Source: PLoS One. 2013 Apr 4;8(4):e61298. doi: 10.1371/journal.pone.0061298 (PMC3617180; doi:10.1371/journal.pone.0061298)
Supplement: Table S5 — Summary of classification results of Canonical Variates Analysis (CVA) of squirrel mandible shape using the locomotion categories of Michaux et al . [16] as grouping variable. The fossil Douglassciurus jeffersoni was assigned to the arboreal/scansorial group with p = 0.816. (DOCX) [file pone.0061298.s010.docx]

**Table S5. Summary of classification results of Canonical Variates Analysis (CVA) of squirrel mandible shape using the locomotion categories of Michaux *et al.* [16] as grouping variable.** The fossil *Douglassciurus jeffersoni* was assigned to the arboreal/scansorial group with *p* = 0.816.

|  | Gliding | Arboreal/Scansorial | Terrestrial |
| --- | --- | --- | --- |
| Gliding | 98.1  (94.2) | 1.9  (5.8) | 0.0  (0.0) |
| Arboreal/Scansorial | 1.5  (3.0) | 94.1  (91.1) | 4.4  (5.9) |
| Terrestrial | 0.0  (0.9) | 14.0  (15.8) | 85.1  (83.3) |

The numbers refer to the percent of cases assigned to each category. The results after cross-validation are in brackets.
